# Interrelated Roles of Chloride and Bicarbonate in Regulating Electron Transport Across Photosystem II in *Limnospira maxima*

**DOI:** 10.3390/plants15101490

**Published:** 2026-05-13

**Authors:** Leslie Castillo, Nicole Seliga, Nidhi Patel, Grant Steiner, Gustavo Chavez, Alexis Diaz, Colin Gates

**Affiliations:** 1 Department of Chemistry and Biochemistry, Loyola University Chicago, Chicago, IL 60660, USAnseliga@luc.edu (N.S.); gchavez6@luc.edu (G.C.);; 2 Department of Bioinformatics, Loyola University Chicago, Chicago, IL 60660, USA

**Keywords:** photosystem II, bicarbonate, chloride, bromide, water oxidation, proton-coupled electron transfer

## Abstract

Efficient charge separation and electron transfer in Photosystem II (PSII) depend on small inorganic cofactors that maintain redox balance and catalytic stability. Chloride facilitates water-oxidizing-complex turnover and minimizes charge recombination. Bicarbonate, coordinated to the non-heme iron, facilitates electron transfer between the plastoquinones Q_A_ and Q_B_. This work investigates cooperativity between these cofactors across PSII in the hypercarbonate-requiring cyanobacterium *Limnospira maxima*. Bromide-for-chloride substitution induces a distinct kinetic limitation at the water oxidizing complex. While bicarbonate depletion inhibits electron transfer at the acceptor side, bromide-substituted cells maintain a measurable level of electron flow through the intersystem chain. The presence of bromide induces structural changes that allow partial electron transfer to continue even in the absence of the bicarbonate cofactor, which is not observed in the chloride system. However, this dual anion stress results in irreversible functional impairment in some centers, whereas full recovery of activity is observed with native chloride. When the donor side is restricted by bromide, the loss of bicarbonate, which is thought to function as a proton buffer for the donor side, compromises the overall stability of the reaction center. This leads to a permanent decrease in activity of the electron transfer chain, suggesting an interdependence between the roles of chloride and bicarbonate that is essential for protecting PSII during ionic stress.

## 1. Introduction

Oxygenic photosynthesis is the primary engine of the biosphere, converting sunlight energy into chemical energy while generating nearly all atmospheric molecular oxygen [1,2]. This process is powered by Photosystem II (PSII), a multi-subunit pigment protein complex that performs the light-driven oxidation of water and reduction of plastoquinones [3,4]. As the only biological catalyst capable of splitting water, PSII captures photons and converts this energy into a stable-charge separated state at the P680 reaction center [5]. This generates the oxidizing power necessary to drive the water oxidizing complex (WOC), a Mn_4_CaO_5_ cluster located on the lumenal side of the complex. The WOC cycles through five intermediate oxidation states (S_0_ to S_4_; S_4_ is transient), known as the Kok cycle [6,7]. Each light-induced transition is driven by the removal of an electron by the redox-active D1-tyrosine-161 radical (Y_z_) and is coupled with the sequential release of protons to the lumen [8]. The efficiency of these proton-coupled electron transfer (PCET) steps is regulated by inorganic cofactors in the surrounding environment [9,10]. The presence of essential cofactors, such as chloride (Cl^−^) and bicarbonate (HCO_3_^−^), is required to facilitate the charge balance and proton delivery pathways that allow the Kok cycle to successfully advance through the higher oxidation states [11,12,13,14,15,16,17].

Efficient water oxidation requires the coordination of redox chemistry with rapid proton transfer to prevent destructive charge recombination [18]. During the S-state cycle, protons are released in the S_0_ → S_1_, S_2_ → S_3_ and S_3_ →S_4_ → S_0_ transitions with a stoichiometry of 1:0:1:2 (excluding S_1_ → S_2_) [12,19,20]. This release pattern is attributed to buffering effects from nearby residues that modulate proton transfer and maintain the stability of charge separation [21,22]. This transfer is facilitated by hydrogen bond via a Grotthuss mechanism, where protons are transferred through pathways of ordered water molecules [23,24]. High-resolution structural models, including the 1.93 Å cryo-electron microscopy (cryo-EM) structure of *Synechocystis* sp. PCC 6803 [25], reveal that the Mn_4_CaO_5_ cluster is accessed by three conserved water channels: the O1 “large” channel, the O4 “narrow” channel, and the Cl1 “broad” channel [26]. The WOC region contains two conserved chloride binding sites, Cl1 and Cl2, which are necessary for donor side regulation [12]. The Cl1 site is located within 7 Å from the manganese cluster and is coordinated by the side chains of D1-N181 and D2-K317 [6,27]. It is postulated to act as a gatekeeper, maintaining the hydrogen bonding networks necessary for proton egress toward the lumen. By stabilizing the local environment, Cl1 facilitates the advancement of the Kok cycle during the higher S-state transitions and prevents the formation of inhibitory salt bridges [27,28,29]. The Cl2 site is approximately 12 Å from the “narrow” O4 channel [6]. While its role is less defined, Cl2 is thought to contribute to structural stability and may influence substrate water delivery [27]. The specificity of these sites is demonstrated by anion substitutions; while monovalent anions like F^−^ and NO_3_^−^ act as inhibitors that impede proton release and delay S-state cycling, Br^−^ preserves function but introduces kinetic delays [30,31,32,33,34]. These delays highlight how the binding site’s chemical properties dictate the catalytic turnover of PSII. However, chloride is only one component of the inorganic cofactor environment, as bicarbonate also plays a role in modulating these electron and proton pathways.

In addition to the chloride requirement, PSII function is dependent on bicarbonate. The well-known “bicarbonate effect” occurs on the acceptor side, where bicarbonate acts as a bidentate ligand to the non-heme iron (NHI) located between the primary electron acceptor Q_A_ and the secondary, exchangeable quinone Q_B_ [11,35]. High-resolution crystallography and cryo-EM studies have confirmed this coordination, where bicarbonate is anchored by a hydrogen bonding network involving residues such as D1-R257, D1-H252, D2-Y244 and D1-Y246 [6,36,37,38,39]. At this site, the ligand stabilizes the protein environment and fine tunes the redox potential between the primary and secondary quinones to ensure efficient linear electron transfer [40,41,42]. Additionally, this local hydrogen bonding environment is thought to facilitate the specific protonation events required for the reduction of plastoquinone (PQ) at Q_B_ to PQH_2_ [36,37,43]. Beyond this fixed coordination at the NHI, studies point toward a significant but dynamic role on the donor side [11,44,45]. Unlike the well-defined chloride binding sites within proton egress channels, structural maps have yet to resolve a static bicarbonate site near the WOC. Instead, HCO_3_^−^ is suggested to associate dynamically with positively charged residues such as CP43-R357 to operate as a mobile proton carrier [46,47]. In this capacity, bicarbonate may diffuse through the channel’s hydrogen bonding networks to neutralize protons released during water oxidation [44,48,49,50]. Additionally, bicarbonate is necessary for photoassembly and repair of the Mn_4_CaO_5_ cluster, where it is thought to electrostatically steer Mn^2+^ ions toward the catalytic site [17,51,52]. To explore these roles, researchers utilize inhibitors like formate (HCO_2_^−^) for bicarbonate depletion, providing a way to study how the absence of this buffer alters the kinetics of the Kok cycle [53,54,55].

The cyanobacterium *Limnospira maxima* (*L. maxima*) is a unique system for exploring the relationship between these inorganic cofactors. Adapted to extreme environments of alkaline soda lakes with a pH of 10–11, *L. maxima* thrives in bicarbonate concentrations as high as 1.2 M, conditions that would be inhibitory to most other phototrophs [45,46,56]. Along with this tolerance, the organism has evolved a specialized photosynthetic system that is characterized by the unique kinetic behavior of its PSII [46,57,58]. In most phototrophs, the rate-limiting step is associated with the acceptor side due to the slow turnover rate of the plastoquinone pool [59]. However, *L. maxima* exhibits fast acceptor side kinetics, with a Q_B_ site turnover frequency of at least 500 Hz, which shifts the catalytic limitation to the donor side [57,60]. This makes the WOC sensitive to stress in its local environment. Consequently, *L. maxima* is an appropriate model system for investigating how cofactors like chloride and bicarbonate coordinate to maintain high efficiency under environmental stress, providing a look into donor side regulation that may be hidden in other photosynthetic organisms.

Despite the well-established structural role of bicarbonate on the acceptor side, its potential as a dynamic regulator of the donor side remains a critical gap in our understanding of the photosynthetic electron transport chain. Existing species variation adds a layer of complexity to this role, as the reported extent of the donor side “bicarbonate effect” varies across different organisms [11,61]. Because both bromide substitution and bicarbonate depletion independently affect the efficiency of S-state advancement and proton egress, this study investigates the degree of interdependency between these two ionic cofactors [34,62]. This work examines how bicarbonate depletion impacts PSII activity in bromide-substituted cells compared to the native chloride-containing system. By characterizing this interaction, this study aims to clarify anionic cofactor requirements for stabilizing water oxidation and sustaining rapid turnover of the water oxidizing complex.

## 2. Results

### 2.1. Rate Oximetry

A comparative analysis of oxygen evolution rates in native (Cl^−^) and bromide-substituted *L. maxima* was conducted to investigate the role of the halide cofactor in the bicarbonate mediation of PSII activity (Table 1). A full depletion and repletion method was utilized to assess the functional resilience of the water oxidizing complex under both halide conditions. Under growth light (25 µmol photons m^−2^ s^−1^), native chloride cells exhibited an oxygen production rate of 113.25 µmol O_2_ mg Chl *a*^−1^ h^−1^. The substitution of Cl^−^ with Br^−^ resulted in a 6% decrease in initial steady-state activity (106.27 µmol O_2_ mg Chl *a*^−1^ h^−1^). The effectiveness of the bicarbonate depletion protocol was confirmed by a significant reduction in oxygen evolution across all samples. However, a difference in sensitivity to bicarbonate removal was observed; bromide-substituted cells exhibited a 71.35% loss of activity, whereas native cells showed a 59.20% reduction in activity post depletion. This increased inactivation suggests a lower functional stability of the bromide-substituted WOC when the bicarbonate cofactor is removed. Another distinction between the halide treatments was observed during the repletion phase. Upon the re-introduction of bicarbonate, native Cl^−^ cells achieved a full recovery to 104.3% activity, indicating that the depletion protocol does not induce irreversible damage to the PSII complex. In contrast, Br^−^ substituted cells exhibited a persistent recovery maximum, returning to only 82.68% of the original baseline. This disparity suggests that Br^−^ substitution impairs the functional return of the bicarbonate cofactor.

### 2.2. Chlorophyll Variable Fluorescence Kinetics

**Figure 1:**
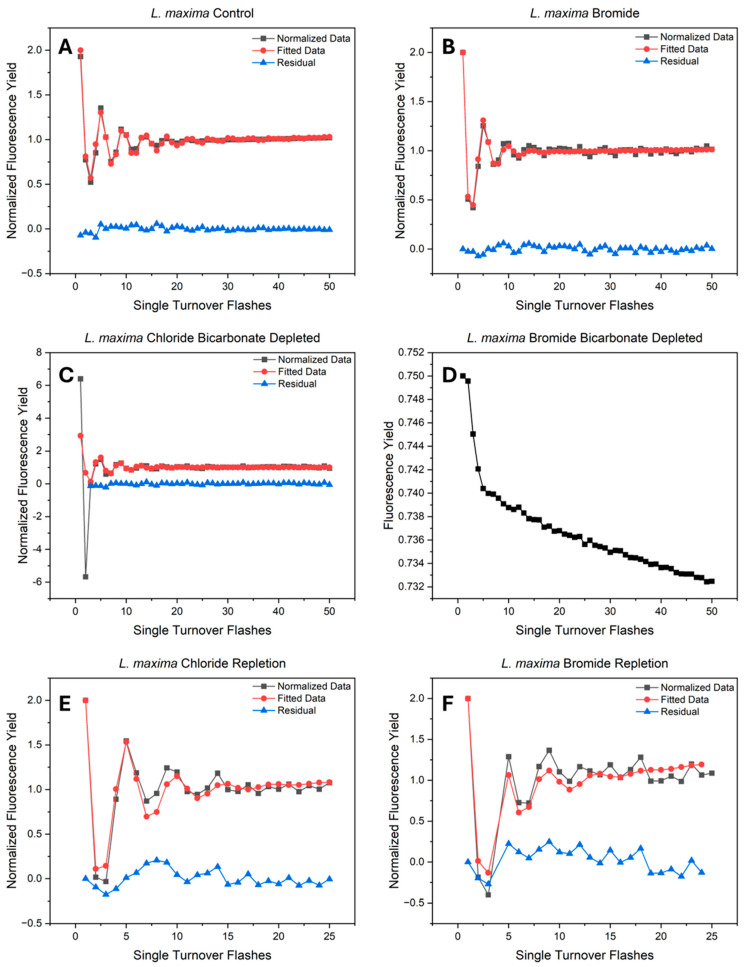
Flash induced F_v_/F_m_ oscillations under varying halide and bicarbonate conditions. Panels represent (**A**) chloride control, (**B**) bromide-substituted, (**C**) chloride/bicarbonate-depleted, (**D**) bromide-substituted/bicarbonate-depleted, (**E**) chloride/bicarbonate-repleted, and (**F**) bromide/bicarbonate-repleted conditions. All samples were exposed to a train of 50 saturating single-turnover flashes (STFs) to induce S-state transitions. Data are representative of at least 5 biological replicates.

Fast Repetition Rate (FRR) fluorometry was employed to assess the kinetics of the assembled photosystems using a series of single turnover flashes (STFs) to advance the complex through its sequential oxidation states. The resulting period-four oscillations (Figure 1) map the stepwise oxidation progression of the Mn_4_CaO_5_ cluster. By fitting these oscillations to the VZAD model, inefficiency parameters and initial S-state populations were derived (Table 2). In the native control cells, characteristic period-four variable fluorescence oscillations were observed (Figure 1A). These peaks are obtained through synchronized S-state WOC cycling, driven by efficient charge separation and forward electron transfer. While both halide environments supported baseline periodicity, the bromide-substituted cells (Figure 1B) displayed reduced oscillation amplitudes and faster dampening than the chloride control, reflecting lower WOC efficiency due to bromide substitution. However, bicarbonate depletion caused disruptions to this periodicity, where the kinetic failure suggests being dependent on the halide environment. In the depleted chloride cells (Figure 1C), the quality of oscillations is diminished compared to the native control. Visible oscillations remain at a much lower intensity, although there is a scaling change after the second flash. Notably, the VZAD fit for this state was applied only to flashes from the third onward, with the first two flashes modeled to reflect expected behavior, consistent with the rest of the flash train. However, bicarbonate depletion in bromide-substituted cells (Figure 1D) eliminated period four oscillations, resulting in decay in fluorescence yield. Although bicarbonate depletion primarily limits the acceptor side at the non-heme iron, this result suggests that the bromide-substituted water oxidizing complex cannot advance through the S-states against the backup of electrons at the acceptor side.

Reintroducing bicarbonate restored period-four oscillations in both halide conditions (Figure 1E,F), demonstrating the reversibility of the acceptor side bottleneck and the reactivation of the water oxidizing complex. However, the repleted signals exhibited increased noise and an increased root mean square error (RMSE) during modeling compared to the baselines. Consequently, the VZAD fitting range was restricted to the first 25 flashes to minimize residual error and ensure accurate parameterization. Analysis of the S-state transition revealed that while cycling was restored, with the WOC operating with altered kinetics (Table 2). Specifically, the alpha parameter, representing the probability of a “miss” or failure to advance to the next S-state, increased in the repleted samples compared to their native controls. Chloride miss rates increased from 0.125 to 0.205, and for bromide substitution from 0.179 to 0.194. However, the double hit parameter (beta), which originally increased to 0.096 during chloride bicarbonate depletion, returned to 0.00 in both repleted conditions. Repletion induced a redistribution of the resting S-state populations toward higher oxidation states. While the native cells maintained a standard distribution across the reduced S_0_ and S_1_ states, the repleted cells exhibited a loss of the S_0_ population. Instead, the centers showed an accumulation in the oxidized S_2_ state, which accounted for 0.581 of the population in repleted chloride and 0.541 in repleted bromide. This indicates that while the WOC is functional, bicarbonate stress results in an oxidized resting state relative to the native cells.

**Figure 2:**
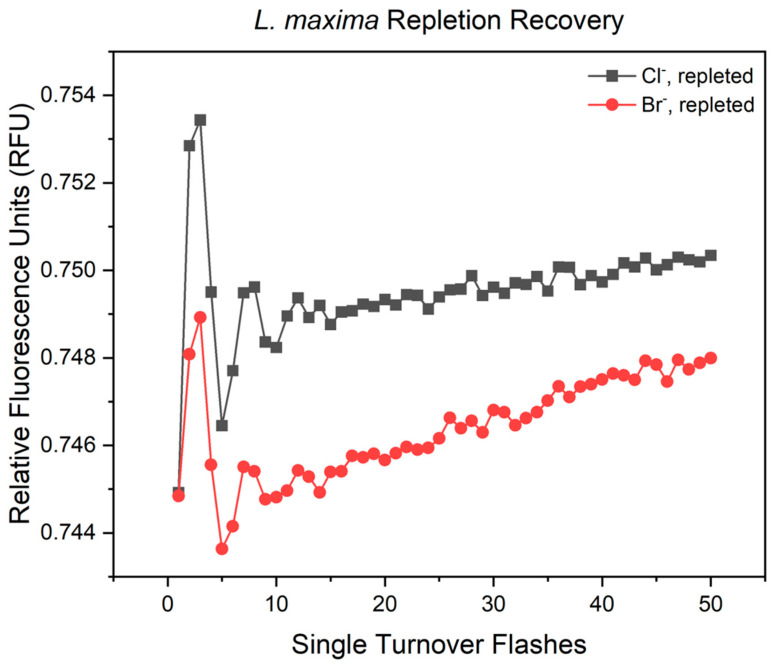
Recovery of PSII electron transport following bicarbonate repletion in chloride native versus bromide-substituted *L. maxima*. Single-turnover flash-induced fluorescence was measured across a sequence of 50 flashes. Cells were initially depleted of bicarbonate and then reintroduced to bicarbonate as described. Data are representative of 5 biological replicates.

Following the analysis of the S-state distribution and cycle efficiency, the overall capacity of the water oxidizing complex was quantified by evaluating the variable fluorescence yield oscillation amplitudes (Figure 2). According to the Kok model, dark adapted photosystems predominantly rest in the S_1_ state. The third flash (Y_3_) drives the largest population of centers through the S_3_→S_4_→S_0_ transition, producing the maximum amplitude in fluorescence yield, which correlates with the concurrent oxygen release. The fifth flash (Y_5_) corresponds to the minimum in oxygen evolution. The amplitude between these two points (Y_3_–Y_5_) provides a measurement of the proportion of active PSII centers advancing through the S state cycle. In the chloride-repleted system, the oscillation amplitude was measured at 0.00698 RFU. In the bromide-repleted system, the oscillation was reduced, resulting in an amplitude of 0.00530 RFU. This amplitude indicates that upon the reintroduction of bicarbonate, the bromide-substituted photosystems recover approximately 76% of the active S-state cycling of the chloride cells. This reduction in yield demonstrates that the elevated inefficiency parameters reflect a physical depletion of active centers.

### 2.3. Q_A_^−^ Reoxidation

To investigate the interplay between inorganic cofactors on the acceptor side of PSII, Q_A_^−^ reoxidation kinetics were measured in cells where the native halide (Cl^−^) was substituted with bromide and cells were subsequently depleted of bicarbonate. This approach allows for a direct evaluation of how anion identity influences the stability and function of the Q_B_ binding site when bicarbonate availability is limited. To measure these electron transfer kinetics, the decay of variable fluorescence was fitted to a biphasic exponential model where each parameter resolves a functional state of the PQ binding site. The Y_0_ value represents the fraction of centers that are either functionally inactive or unoccupied, meaning they lack a plastoquinone molecule in the Q_B_ site at the time of the flash and cannot undergo forward electron transfer. The fast phase, defined by amplitude A_1_ and time constant t_1_, describes the primary electron transfer from Q_A_^−^ to a pre-bound Q_B_ molecule. Here, A_1_ corresponds to the proportion of centers undergoing this transfer, while t_1_ indicates the rate of the reaction, occurring in the microseconds range. The parameters A_2_ and t_2_ reflect the second electron transfer step from Q_A_^−^ to a semi-reduced Q_B_^−^. This phase is slower, with a t_2_ value in the milliseconds range, as the process is often rate-limited by the protonation steps required to stabilize the double reduction of quinone into plastoquinol.

**Figure 3:**
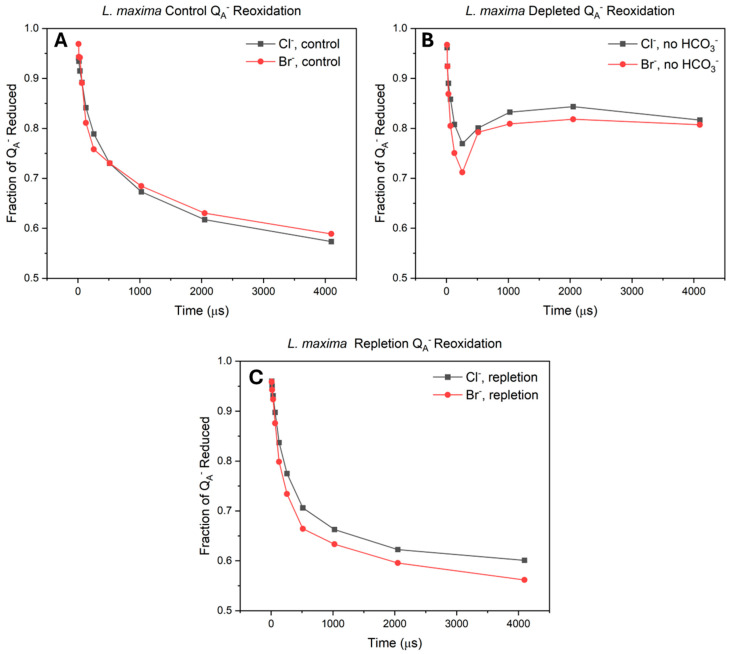
Biphasic exponential decay curves of Q_A_^−^ reoxidation across halide and bicarbonate treatments. Panels represent (**A**) control, (**B**) bicarbonate-depleted, and (**C**) bicarbonate-repleted conditions, comparing cells grown in native chloride and bromide-substituted environments. Data points represent the average of at least 4 biological replicates obtained using variable pump-probe flashes to measure the reduced fraction of Q_A_^−^. Representative fits to data are shown in Figure S1.

As shown in Figure 3A and Table 3, the native control (Cl^−^ baseline) displayed a fast phase of electron transfer (t_1_) of 158 µs, with an amplitude (A_1_) of 0.158, representing the proportion of centers undergoing this electron transfer event. The slower transfer phase from Q_A_^−^ to Q_B_^−^ (t_2_) was recorded at 1420 µs with an amplitude (A_2_) of 0.234, while the fraction of inactive centers (y_0_) was 0.561. The bromide substitution resulted in an acceleration of this initial step, with the t_1_ lifetime decreasing to 103 µs. Alongside this increased rate, the amplitude A_1_ increased to 0.220, suggesting that the presence of bromide not only influences the kinetic rate of Q_A_^−^ reoxidation but also increases the proportion of PSII centers undergoing rapid electron transfer to the plastoquinone pool. While the fast phase was accelerated, the slow phase rate was 1850 µs and resulted in an amplitude of 0.207 (A_2_), suggesting that bromide influences the primary and secondary electron transfer steps differently. The baseline Y_0_ for bromide remained nearly identical to the control.

Upon bicarbonate depletion, a 5-flash fit was employed because the resulting decay curves became irregular to fit the standard biphasic model (Figure 3B). This loss of model fit was consistent across both chloride and bromide-containing cells. The A_2_ and t_2_ phases were absent, indicating that exponential decay ceased after the initial fast phase. In Cl^−^ depleted cells, the t_1_ value decreased to 73.0 µs, while Br^−^ depleted cells showed an even greater acceleration, with a t_1_ of 53.0 µs. During this state, y_0_ values increased to 0.767 (Cl^−^) and 0.716 (Br^−^). Although electron transfer is initiated, the abnormal kinetic profile and the failure to reach Q_B_^−^ suggests that the Q_B_ site cannot achieve full turnover without bicarbonate. This results in a limitation on the acceptor side, even though the primary transfer rate is still sensitive to the halide substitution. To determine if bicarbonate depletion caused lasting damage to PSII, Q_A_^−^ reoxidation was also measured following the reintroduction of bicarbonate (Figure 3C). In the repleted cells, the kinetics were restored to a biphasic model, confirming that the integrity of the complex was maintained. For chloride repletion, y_0_ was measured at 0.596, with a t_1_ value of 171 µs, an A_1_ value of 0.220, and a restored slow phase of t_2_ 1160 µs. Similarly, Br^−^ repleted cells showed a return to biphasic behavior, with a y_0_ of 0.543, a t_1_ of 153 µs, an A_1_ of 0.286, and a t_2_ of 1970 µs with an amplitude of 0.149 (A_2_). The recovery of these inactive centers and kinetics suggest that the inhibitory effect of bicarbonate depletion is fully reversible. Additionally, the data emphasizes that while halide identity modulates the rates of electron transfer, the fundamental turnover of the Q_B_ site remains regulated by the presence of bicarbonate and non-heme iron.

### 2.4. Redox Kinetics of the Cytochrome b_6_f Complex and Plastocyanin

**Figure 4:**
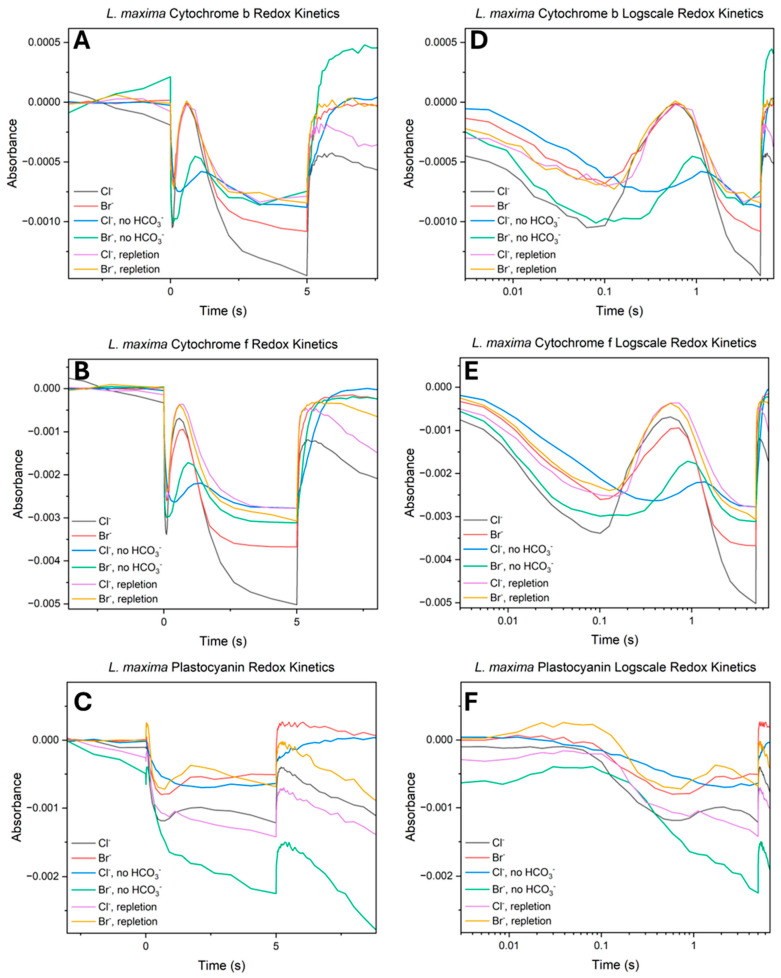
Redox kinetics of cytochrome b_6_f and plastocyanin across halide and bicarbonate treatments. Panels represent linear time-scale kinetics for (**A**) cytochrome b, (**B**) cytochrome f, and (**C**) plastocyanin, with corresponding log-scale kinetics shown in panels (**D**–**F**). Negative absorbance values signify oxidation of the respective centers, while positive values indicate reduction. Each panel compares the six conditions. Data represent the average of at least five independent biological replicates.

The cytochrome b_6_f complex facilitates electron transfer between PSII and photosystem I (PSI) by oxidizing plastoquinol and reducing plastocyanin (PC) or cytochrome *c*_6_ (Figure 4). This complex comprises four redox centers, including the iron-sulfur cluster (Fe_2_S_2_) and the hemes of cytochrome b_6_ (cyt b) and cytochrome f (cyt f). To determine the location of the kinetic limitations observed in bromide- and bicarbonate-depleted cells, the redox transitions of these components were monitored under continuous illumination. In these measurements, the kinetic traces for cyt b and cyt f exhibit an initial decrease in absorbance (trough) upon illumination, signifying the rapid removal of electrons by the P700^+^ reaction center through the PC pool. This oxidation trough represents both the redox poise of the system and the time required to remove electrons, while the subsequent rise (peak) corresponds to the reduction by arrival of electrons from PSII.

The influence of halide substitution and bicarbonate availability on cytochrome b is shown in Figure 4A,D. In native Cl^−^ containing cells, a rapid removal of electrons is observed, signifying the efficient pull of electrons by PSI, which is immediately followed by a robust resupply from PSII that results in a well-defined reduction peak. While Br^−^ bromide-substituted cells maintain this overall kinetic profile, the initial oxidation is shallower, and the amplitude of the resupply peak is noticeably delayed. This suggests a shift in the redox equilibrium and restriction of the electron transport chain even when bicarbonate is present. Bicarbonate removal induces a significant regulatory shift. In chloride containing cells, depletion imposes kinetic constraints, observed by a substantial reduction in the initial oxidation amplitude of PSI and heavily restricted electron resupply from PSII. However, the depleted bromide state appears perturbed by the lack of bicarbonate. During depletion, bromide cells maintain more negative oxidation troughs and exhibit faster, higher amplitude reduction peaks than the chloride cells. This demonstrates that Br^−^ allows a small but continuous residual electron flow to move through the chain when bicarbonate is missing. Despite this higher residual flux during depletion, the Br^−^ system fails to recover as well as the native system. When bicarbonate is repleted, the Cl^−^ cells return to a state similar to their original baseline. In contrast, the Br^−^ cells fail to achieve full restoration, as cyt b remains in a state of lower oxidation amplitude, similar to that observed during depletion (Figure 4A,D).

Similar kinetic trends are observed for cytochrome f (Figure 4B,E), which acts as the intermediate electron donor to the PC pool. In the native Cl^−^ control, cyt f presents an initial oxidation trough followed by a rapid reduction peak, indicating a high volume of electrons arriving from PSII. In the Br^−^ control, this inflow of electrons is already compromised, evidenced by a delayed and slower reduction recovery. During bicarbonate depletion, cyt f mirrors the behavior seen in cyt b: the Br^−^ cells maintain a more active redox profile than the Cl^−^ depleted cells. This further supports the mechanism that Br^−^ allows for greater residual electron flux when bicarbonate is absent. However, upon repletion, cyt f in the Br^−^ system fails to return to the native baseline. The repletion recovery phase remains delayed, suggesting that the kinetic bottleneck identified at the b_6_f complex is not localized to a single heme but reflects a systemic starvation of electrons arriving at the complex.

The redox state of plastocyanin, shown in Figure 4C,F, provides a direct look at the balance between the supply of electrons from the b_6_f complex and the demand from PSI. Because PC transfers electrons directly to the P700 reaction center, its absorbance traces reveal the efficiency of the delivery line. In native Cl^−^ cells, the PC pool is rapidly oxidized upon illumination (peak) and then quickly reduced by incoming electrons from cyt f (trough). In the Br^−^ substituted control, this resupply phase is delayed, consistent with the restriction observed in the b_6_f complex.

During bicarbonate depletion, the behavior of the system shifts. In the chloride-depleted state, the initial oxidation feature is smaller, and the subsequent reduction is nearly flat, suggesting a restriction in electron flow. In the bromide-depleted state, an initial oxidation peak is observed, but the system fails to exhibit a reduction trough during continuous illumination. This sustained oxidation suggests that while a residual flow of electrons moves through the hemes, it is consumed by the demand of PSI and cannot accumulate to reduce the PC pool. When bicarbonate is repleted, the Cl^−^ cells demonstrate high reversibility and return to their original baseline. In contrast, the Br^−^ repleted cells exhibit a distinct kinetic delay. While PSI is able to oxidize the PC pool, the rate at which those electrons are replaced by PSII is slower than in the Cl^−^ control. This persistent delay is visible in the recovery of the log-scale plots.

### 2.5. Redox Kinetics of Photooxidized P700 (P700^+^)

**Figure 5:**
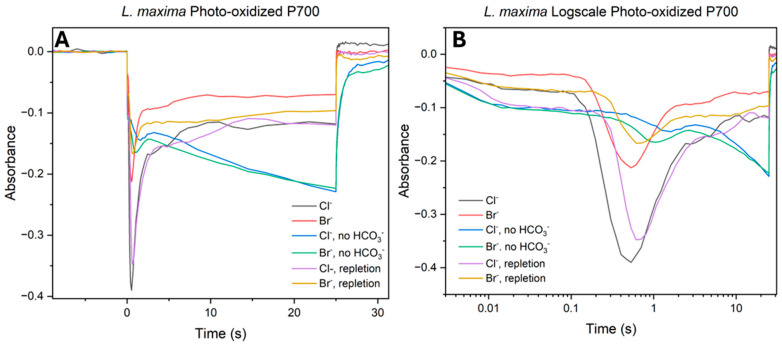
Photo-oxidized P700^+^ absorbance kinetics across halide and bicarbonate treatments. Absorbance measurements were taken at 810 nm to monitor the redox state of the PSI reaction center. Panels represent (**A**) linear time-scale kinetics and (**B**) log-scale kinetics. Each panel compares the six conditions. Data represent the average of at least five independent biological replicates.

To investigate the efficiency of electron transport under halide-substituted conditions, the redox state of the PSI reaction center was measured under continuous light (Figure 5A,B). Monitoring absorbance changes provides an assessment of the kinetic balance between electron consumption by PSI and the resupply from upstream components, specifically PSII. In these kinetic traces, a decrease in absorbance signifies the photooxidation of P700 to P700^+^, while the subsequent peak (reduction) in absorbance characterizes the arrival of electrons originating from PSII. In native chloride cells, initial illumination triggered a rapid and deep photooxidation of P700, characterized by an initial decrease in absorbance. This trough was followed by a secondary feature, a rapid reduction peak signifying the arrival of electrons from an activated PSII. While Br^−^ substituted cells displayed a similar kinetic profile, the maximum amplitude of oxidation was consistently reduced compared to the native system. Similarly, the secondary reduction feature in the Br^−^ control appeared less pronounced, suggesting that Br^−^ substitution shifts the steady-state redox balance of the electron transport chain even in the presence of bicarbonate. The removal of bicarbonate resulted in a kinetic bottleneck across both halide treatments. In Cl^−^ depleted cells, the reduction peak was suppressed, replaced by a slow and linear recovery phase, which suggests a significant delay in downstream electron delivery by PSII. This effect was significant in Br^−^ substituted cells; the bromide depletion trace exhibited both a shallower oxidation phase and an attenuated reduction peak relative to the native depleted cells. This suggests a limitation on the donor side electron flux; likely the activation of the WOC is not entirely halted but turnover is occurring at a rate insufficient to match the initial oxidative demand of PSI. The repletion of bicarbonate highlighted the functional difference between the two halides. Native Cl^−^ centers showed near reversibility, with the repleted traces closely similar to the control in both the amplitude of the oxidation trough and the timing of the reduction peak. However, Br^−^ substituted cells did not attain full kinetic restoration. Although the addition of bicarbonate improved electron flux compared to the depleted state, the Br^−^ repletion traces remained kinetically delayed. The recovery of the reduction peak was incomplete, and a persistent lag was observed in the arrival of electrons from PSII. This kinetic delay correlates with the deficit observed in previous measurements.

### 2.6. Fluorescence Emission Spectroscopy

**Figure 6:**
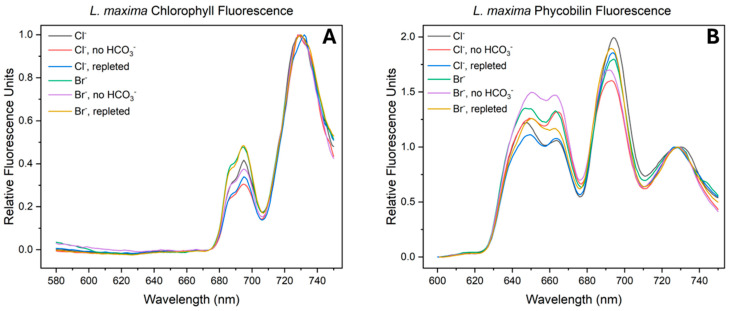
The 77 K fluorescence emission spectra of *Limnospira maxima* across halide and bicarbonate treatments. (**A**) Chlorophyll excitation at 435 nm and (**B**) phycobilin excitation (561 nm) spectra of cells grown in either native chloride or bromide-substituted media. For each halide condition, three conditions are compared: control (bicarbonate present), bicarbonate-depleted, and bicarbonate-repleted. Spectra are representative of at least six biological replicates (*n* = 6). To facilitate comparison of energy distribution, all spectra were normalized to the PSI emission peak (~730 nm). Individual pigments (CPC, APC, F685, F695 and PSI) were resolved via Gaussian deconvolution, as shown in Figures S2–S13 and Tables S1–S12.

The structural arrangement of the photosystems and the distribution of excitation energy were measured by 77 K fluorescence emission spectroscopy, utilizing direct excitation of chlorophyll at 435 nm and phycobilin at 561 nm. This measurement allows for the identification of the two primary PSII internal subpeaks: F685 (CP43), often associated with the reaction center, and F695 (CP47), the “chlorophyll trap”. For the purpose of ratio calculations, the total PSII emission was defined as the integrated area of both the F685 and F695 subpeaks. As shown in Figure 6A and Table 4, halide identity and bicarbonate availability modulate the structural organization within the thylakoid membrane. In the native Cl^−^ control, the PSII:PSI ratio was measured at 0.270, with a corresponding F685:F695 ratio of 0.235. Bromide substitution resulted in an increase in both parameters, yielding a PSII:PSI ratio of 0.311 and an F685:F695 ratio of 0.356. The increase in the PSII:PSI emission ratio suggests a greater amount of chlorophyll associated with PSII relative to PSI. This change in distribution is consistent with a shift toward a higher relative abundance of PSII. Additionally, the elevated F685:F695 ratio in the Br^−^ cells suggests a greater amount of chlorophyll associated with the PSII reaction center compared to native Cl^−^ control.

Bicarbonate depletion caused a substantial decrease in the PSII:PSI ratio across both halide conditions (0.193 for Cl^−^ and 0.249 for Br^−^), indicating a loss of structural connectivity to PSII relative to PSI. The F685:F695 ratio increased to 0.381 for the Cl^−^ depleted sample and 0.447 for the Br^−^ depleted sample. In the context of overall lower PSII populations, this energy accumulation at F685 likely indicates structural decoupling or a physical limitation in energy transfer from CP43 to the reaction center when bicarbonate is absent. The repletion of bicarbonate facilitated a recovery of photosystem stoichiometry, particularly in the bromide-substituted cells. In Br^−^ repleted cells, the PSII:PSI ratio returned to 0.299, nearly reaching baseline levels (0.311). The Cl^−^ repleted cells only recovered to a ratio of 0.209, remaining much lower than the native control. The F685:F695 ratios followed a similar trend of recovery; while the Cl^−^ repleted sample reached a value of 0.447, the Br^−^ repleted sample returned to 0.308, closer to its original baseline. This suggests that bromide facilitates the restoration of energetic coupling between the CP43 and CP47 inner antennas and the reaction center, restoring efficient energy transfer.

To observe the balance of exciton flow through the external antenna complexes and their structural association with the photosystems, 77 K fluorescence emission spectra were measured using phycobilin excitation at 561 nm. The resulting emission spectrum is characterized by two distinct subpeaks: a shorter-wavelength, higher-energy peak attributed to c-phycocyanin (CPC) and an adjacent lower-energy peak corresponding to allophycocyanin (APC). For these ratio calculations, the total phycobilin (PB) emission was defined as the sum of the area of the CPC and APC subpeaks, while the photosystem (PS) emission represents the sum of the PSII (F685 and F695) and PSI area. By evaluating the CPC:APC, F685:F695, PSII:PSI, and PB:PS emission ratios, the efficiency of energy transfer from the peripheral antenna to the reaction center core can be monitored.

In the native chloride environment, *L. maxima* exhibited a CPC:APC ratio of 1.31, while bromide substitution resulted in a notably lower ratio of 1.10. This decrease suggests that bromide facilitates energy transfer from the peripheral CPC rods toward the terminal APC emitters within the phycobilisome. This coupling is also reflected in the PSII state, where the baseline F685:F695 ratio was significantly lower in bromide cells (0.089) compared to the chloride control (0.140). This lower ratio reflects inefficient energy transfer from the antenna to the reaction center, and the accumulation at F695 suggests a baseline level of energy trapping at CP47 induced by the bromide substitution. The PB:PS ratio serves as a measurement for the physical coupling between the antenna to the core and the extent to which excitation energy fails to transfer to the reaction centers. In the control samples, the PB:PS ratio was 0.572 for chloride and 0.720 for bromide. This suggests that while bromide modifies internal antenna efficiency and core coupling, it may support a higher population of uncoupled or “free” phycobilisomes at the baseline, resulting in higher emissions. Under baseline conditions, the distribution of energy from the phycobilisome to the individual photosystems (PSII:PSI) shifted from 0.928 in the chloride control to 0.955 in the bromide-substituted cells. This minor increase in energy transfer to PSII aligns with the results seen at 435 nm; because bromide substitution induces a larger overall population of PSII centers, a proportionally greater number of antenna complexes structurally couple to PSII.

Bicarbonate depletion triggered a structural uncoupling between the antenna and the reaction centers across both halide conditions. As seen in Table 5, the CPC:APC shifted to 1.14 for both chloride and bromide. However, the internal PSII state showed a different response to this stress. In chloride-depleted cells, the F685:F695 ratio decreased to 0.121, while the PB:PS ratio increased significantly to 0.738. The rise in phycobilin (PB:PS) emission suggest a physical detachment, where excitation energy is likely failing to reach the reaction center. In contrast, bromide-depleted cells exhibited a dual stress response, where the F685:F695 ratio doubles to 0.179 alongside a PB:PS increase to 0.809. While the elevated PB ratio suggests that a fraction of the phycobilisome dissociates, the increase in F685 indicates that for the population of antennas that remain coupled, forward energy transfer is restricted at the proximal antenna. Within these intact complexes, excitation energy accumulates and fluoresces from CP43 rather than transferring forward. The distribution of energy between the photosystems (PSII:PSI) similarly shifted during depletion, moving to 1.00 for chloride and 1.01 for bromide. This increase in PSII emission contrasts with the observed data at 435 nm, where the population of PSII decreased relative to PSI. This difference suggests that while the abundance of PSII centers declined under stress, the remaining complexes exhibit an increased fluorescence. Because there is a limitation at CP43 preventing excitation energy from entering the reaction center, the built-up energy is forced to be re-emitted as fluorescence, thereby increasing the emission despite the loss of PSII centers.

To determine the level of recovery, the reintroduction of bicarbonate to depleted cells was monitored. As shown in Table 5, the CPC:APC ratio returned to 1.07 for chloride and 1.06 for bromide. Notably, while the bromide-repleted sample returned to its baseline (1.10), the chloride-repleted sample dropped to significantly lower than its original control (1.31). The overall coupling of the antenna (PB:PS) was 0.607 for Cl^−^ and 0.643 for Br^−^. While the chloride-repleted cells retained a slightly higher proportion of uncoupled antenna compared to the native baseline (0.572), the bromide-repleted cells achieved a lower ratio than their original baseline (0.720). This suggests that the stress and recovery induces a physical coupling of the phycobilisome to the core complexes. Additionally, the distribution of energy between the photosystems (PSII:PSI) was altered. The repleted ratios were measured at 1.02 for chloride and 1.06 for bromide, both remaining higher than their controls (0.928 and 0.955). This indicates that repletion promotes a slightly higher association of the antenna with PSII than initially seen even after stress. However, the internal distribution of energy transfer within PSII (F685:F695) reveals that this structural reattachment does not restore function. In the bromide-repleted cells, the ratio decreased back down to 0.084, nearly reaching the initial baseline of 0.089. This decrease indicates that the accumulation of energy at CP43 observed during depletion was resolved. However, the return to this low ratio signifies that the excitation energy is once again sequestered at the CP47 chlorophyll trap. Interestingly, the chloride-repleted ratio dropped to 0.104, resulting in a lower ratio than the original baseline (0.140), indicating that excitation energy transfers forward from the antenna but becomes trapped at the terminal sink.

## 3. Discussion

### 3.1. Donor Side Limitations and Structural Gating

Bicarbonate depletion in bromide-substituted *L. maxima* cells eliminates period-four oscillations in chlorophyll *a* variable fluorescence, indicating an inhibition of the water oxidizing complex. This loss reveals an interrelated dependency between chloride structural gating and bicarbonate chemical buffering. Under native conditions, chloride binds at the Cl1 site near the Mn_4_CaO_5_ cluster to maintain an open proton egress pathway to the lumen [27,63]. Specifically, chloride prevents formation of an inhibitory salt bridge between D1-D61 and D2-K317 residues, maintaining the hydrogen bonding network necessary to shuttle protons and avoid destructive charge recombination during water oxidation [28,29,64]. Although bromide occupies the Cl1 binding site, its larger ionic radius and softer Lewis base properties structurally modify the environment and create a kinetic bottleneck [34,64]. As demonstrated by the bromide-substituted baseline, and consistent with previous observations of halide substitution [34,65], this difference slows the S_2_→S_3_ and S_3_→S_0_ transitions and delays proton release, though the WOC is still able to advance electrons [34]. Bicarbonate is proposed to function as a mobile proton carrier that diffuses through the protein environment to buffer protons generated during water oxidation [15,46,48], serving as a dynamic chemical buffer that complements the fixed chloride-dependent egress pathway [6,15,16,27,44,48]. In bicarbonate-depleted bromide cells, dual inhibition occurs, as bromide sterically blocks proton egress and limited bicarbonate prevents local buffering [34]. Without bicarbonate to dissipate the proton gradient, the hindered WOC is unable to advance S-state transitions. This combined effect demonstrates that the native chloride ion is not just an electrostatic placeholder, it provides structural gating that operates with bicarbonate’s buffering capacity to facilitate efficient water oxidation.

Further evidence for this dependency is seen in the recovery kinetics of oxygen evolution following bicarbonate repletion. Because bicarbonate binding is normally reversible [46,66,67,68,69], native chloride-containing cells fully restore and even slightly exceed their baseline oxygen evolution capacity upon repletion (achieving 104.32% recovery). This recovery indicates that chloride accommodates the dynamic rebinding of bicarbonate without permanent structural damage to the WOC. In contrast, bromide-substituted cells exhibit a persistent deficiency, reaching a maximum recovery of 82.68%. This reduced restoration suggests that dual anion stress imposes a more permanent effect on the donor side in the absence of chloride. This overall loss of activity is directly seen by the underlying S-state kinetics. When using the Y_3_–Y_5_ variable fluorescence yield to track the active fraction of centers, the chloride system fully restores its amplitude before bicarbonate depletion. Conversely, the bromide-substituted system recovers only 76% of its active WOC population compared to the native state. This confirms that the loss in gross oxygen evolution is driven by an inactivation of a fraction of reaction centers. The bromide ion not only hinders the proton egress channel during turnover, but its incompatibility likely impairs the structural changes required for the bicarbonate binding site to reorganize and coordinate its mobile buffering network [34]. Therefore, the native chloride ion is necessary not just for water oxidation but for preserving the structural dynamics necessary for the WOC to recover from environmental stress.

### 3.2. Acceptor Side Kinetics

Disrupting both chloride and bicarbonate binding sites reveals long-range regulatory effects across the membrane that modulate acceptor-side electron transfer. Under native conditions, forward electron transfer from the primary quinone (Q_A_^−^) to the secondary quinone (Q_B_) exhibits well-characterized biphasic kinetics, where the secondary slow phase (t_2_) is attributed to the transfer of the second electron to the semiquinone (Q_B_^−^), a process rate-limited by structural gating and protonation events [70,71,72]. The extensive “bicarbonate effect” literature establishes that HCO_3_^−^ dynamically interacts near the non-heme iron and the Q_B_ pocket to facilitate the proton-coupled electron transfer events required for semiquinone stabilization and subsequent plastoquinol formation [62,73]. Prior spectroscopic studies demonstrated that the acceptor side is also subject to halide regulation; specifically, chloride competitively binds at the NHI to displace inhibitors like formate and protect the redox environment of the initial electron transfer [74,75]. However, consistent with these established models, our previous work investigating bicarbonate depletion via formate in native chloride-containing cells demonstrated an effect on the secondary slow phase [67,76]. This localized limitation suggests that the structural reorganization and protonation steps associated with the two-electron gate require the mobile buffering capacity of bicarbonate at a distal site near Q_B_, potentially tuning the chemical properties of the pocket as well [77]. We do not observe a significant degree of Q_A_^−^ decay by superoxide production as has been observed in isolated thylakoids [42].

Bromide substitution reveals a long-range kinetic effect independent of this specific acceptor-side limitation. The steric bulk of the ion induces a kinetic bottleneck at the water oxidizing complex, restricting electron flow and maintaining the downstream PQ pool in a more oxidized state. Therefore, the initial electron transfer proceeds rapidly from Q_A_^−^ into an oxidized Q_B_ without the electrostatic repulsion generated by a partially reduced PQ pool [5,34,78]. Fluorescence decay kinetics confirm this acceleration persists across the baseline, dual anion stress, and the recovery phase, indicating that this rate is dictated by the upstream donor side restriction rather than changes at the acceptor side. While the bromide-substituted baseline retains reduction at the PQ pool, bicarbonate depletion eliminates the secondary phase. Bicarbonate provides the proton donor required to stabilize the formed semiquinone; without this buffering the structural reorganization and protonation steps required cannot progress. When bicarbonate is reintroduced, during the recovery phase kinetics return to the bromide-substituted baseline the t_2_ phase recovers, while the initial t_1_ transfer maintains the accelerated rate indicative of donor side limitation. This demonstrates that independent sites regulate electron transfer: the donor side modulates the initial electron transfer rate by altering the PQ pool redox poise, while the distal acceptor side bicarbonate site controls the proton-coupled progression of Q_B_ being reduced. While high-resolution crystallographic models identify the primary chloride binding sites to the donor side [6,25], spectroscopic studies have demonstrated that the acceptor side also exhibits specific sensitivity to halides [74,75]. These results suggest that in this highly alkaline cyanobacterium, chloride may serve as more than a long-range modulator, potentially playing a transient localized electrostatic role at the stromal surface.

### 3.3. Absorbance Redox Kinetics

The redox kinetics of the cytochrome b_6_f complex, plastocyanin and P700 indicate the overall efficiency of linear electron flow across the thylakoid membrane. While fluorescence decay kinetics isolate specific limitations within PSII, measuring these downstream components reveals how PSII donor and acceptor side constraints impact the photosynthetic electron transport chain [79,80]. Baseline redox kinetics demonstrate that substituting chloride with bromide restricts the capacity of linear electron flow. While the initial oxidation of cytochrome b_6_f, PC and P700 proceeds without delay, their reduction amplitudes are significantly reduced, suggesting an electron limited state in these downstream carriers. This limitation stems from the distinct chemical properties of bromide, including its larger ionic radius and adjusted basicity which restricts the efficiency of S-state transitions at the WOC [34]. Consequently, the rate of PQ pool reduction by PSII cannot match the rate of oxidation by PSI. This aligns with established models of donor side limitations, supporting the conclusion that chloride displacement acts as a limitation for steady state capacity [64,75].

Under bicarbonate depletion, the bromide-substituted cells exhibit a higher continuous residual electron flow than the chloride system. Whereas the chloride bicarbonate-depleted cells inhibit linear electron transfer upon removal of the bicarbonate buffer, the bromide-substituted state maintains a more pronounced oxidation amplitude and a distinct reduction phase at the cytochrome b_6_f complex. This sustained flow suggests that structural perturbations induced by bromide at the donor side may propagate to the acceptor side, disrupting the regulatory gating normally maintained by the bicarbonate site [62,81,82]. This potential long-range structural change across the protein appears to enable a fraction of electron transfer to bypass the disrupted gating mechanism and reach PSI [64]. Alternately, there may simply be an acceptor-side role of chloride which is not structurally detected due to preparatory methods. Repletion kinetics in bromide-substituted samples indicate that this dual anion stress results in irreversible damage. Even after bicarbonate is restored, the electron transport chain exhibits a persistent kinetic delay and fails to recover its baseline amplitude. This sustained downstream delay is consistent with a loss of active reaction centers in PSII. When the water oxidizing complex is restricted by bromide substitution and the acceptor side is simultaneously compromised by bicarbonate depletion, the system becomes highly susceptible to donor-side photoinhibition. Consequently, the kinetic limitation observed downstream suggests that dual anion stress leads to irreversible photodamage at the reaction center, decreasing the turnover rate of the electron transport chain.

### 3.4. Antenna Remodeling as a Response to Anion Stress

Various stressors are known to disrupt the balance between light harvesting and electron transport, causing an accumulation of energy excitation that leaves the P680 reaction center vulnerable to photoinduced damage [83,84]. During dual anion stress in bromide-depleted cells, the forward funneling of excitons into the P680 reaction center creates an accumulation of energy. Under these conditions the reaction center becomes vulnerable to photoinduced damage. The cell’s regulatory response to this energetic limitation is the uncoupling of the phycobilisome antenna complex [85]. Across both halide conditions, the PB:PS ratio increases significantly, indicating an energetic detachment of the phycobilisome as a photoprotective mechanism to limit the influx of energy. Concurrently, the internal F685 emission significantly increases. Because F685 (CP43) originates from the terminal emitters of the phycobilisome and is associated with the reaction center [86], this fluorescence increase indicates a structural disruption in excitation energy transfer to the core. By isolating the compromised reaction center, this energetic decoupling actively mitigates the excitation accumulation to protect PSII.

While 561 nm excitation reveals this disruption in excitation energy transfer, direct chlorophyll excitation at 435 nm isolates the physical stoichiometry and structural integrity of the photosystem cores. At 435 nm, dual anion stress induces a decrease in the PSII:PSI ratio across both halide conditions. This deviation between the two excitation wavelengths indicates that while the remaining PSII complexes fluoresce in response to stimulation at 561 nm due to energetic coupling, the actual abundance of functional PSII reaction centers has declined relative to PSI. This shift points toward a down-regulation of photoinhibited PSII complexes as a protective response to sustained excitation accumulation. The interdependency between chloride and bicarbonate becomes evident during the recovery phase, where repletion resolves the acceptor side bottleneck and prompts reassembly. While both cultures’ phycobilisomes reconnect to the core complexes, seen in a decrease in phycobilin emission, this recovery is dependent on the halide present. In the native chloride environment, the complex maintains its energetic uncoupling even after bicarbonate is restored. Despite the bicarbonate-driven reattachment of the antenna, the internal F685:F695 ratio remains elevated. Conversely, the bromide-substituted cells exhibit a different response upon bicarbonate repletion. The decrease in the F685:F695 ratio indicates that excitation energy is directed to the F695 (CP47) “trap” [86]. Because the donor side remains kinetically limited, the reaction centers cannot utilize this energy for stable charge separation. The bicarbonate-driven recoupling of the antenna forces excitons to bypass productive photochemistry and fall into this sink. This dynamic demonstrates a fundamental disconnect: while bicarbonate initiates the structural reattachment of the antenna, the bromide-induced bottleneck prevents functional recovery, resulting in the loss of excitation energy.

## 4. Materials and Methods

### 4.1. Cell Growth and Media Modifications

*Limnospira maxima* (UTEX LB 2342) was sourced from the University of Texas at Austin Culture Collection (UTEX; Austin, TX, USA) and grown at 30 °C in Zarrouk medium [87], utilizing either the standard chloride conditions or a modified version, where chloride was replaced with equimolar bromide. To ensure the total removal of native chloride, bromide-substituted cultures underwent an adaptation period spanning at least nine generations, achieving a cumulative 10^18^-fold dilution. All cultures were maintained in 100 mL flasks under a 12/12 h light/dark regime using white LED illumination (25 µmol photons m^−2^ s^−1^). Harvesting was performed during the mid-logarithmic phase, specifically between 6 and 8 days post-inoculation [67]. Growth was verified by monitoring optical density at 730 nm using a Thermo-Fisher Genesys 10 spectrophotometer (Waltham, MA, USA).

### 4.2. Bicarbonate Depletion and Reconstitution

Bicarbonate was removed from *L. maxima* cells using a wash treatment with 100 mM sodium formate (pH 7.8) in a bicarbonate-free Zarrouk medium [67]. Sodium formate was utilized as an isotonic substitute to maintain osmotic stability while preventing the internal production of CO_2_, owing to its chemical similarity to bicarbonate and its inability to interconvert into gaseous form [88]. The depletion process involved four successive wash cycles: samples were centrifuged at 9000× *g* for 10 min, the supernatant was discarded, and cells were resuspended in the formate-containing reaction mixture [46]. To ensure full depletion, cells were incubated in total darkness for two hours before measurements. For recovery experiments, bicarbonate was reintroduced at its original concentration of 200 mM (18 g/L) via four washes in the respective chloride or bromide growth media.

### 4.3. Oxygen Measurements

Photosynthetic oxygen evolution and respiratory oxygen consumption were quantified using a Clark-type oxygen electrode (Oxygraph+, Hansatech Instruments; King’s Lynn, UK) [89]. The electrode system consists of a platinum cathode and silver anode connected by a 50% KCl electrolyte bridge, shielded by a Teflon membrane and paper spacer. This membrane allows O_2_ diffusion while protecting the electrode from directly interacting with redox-active species. Prior to measurements, a calibration was performed at ambient room temperature utilizing air-saturated water and nitrogen-purged water, accounting for atmospheric pressure. All measurements were conducted at room temperature under constant stirring. Cells were measured directly within their respective media, maintaining the specific pH and bicarbonate/formate concentrations described in Section 4.2. The resulting current, which is directly proportional to the dissolved O_2_ concentration, was recorded. To determine respiration rates, oxygen depletion was monitored in total darkness for five minutes. Subsequently, oxygen evolution was measured by illuminating cells to the growth light intensity (25 µmol photons m^−2^ s^−1^) for an additional five minutes. Data signifies the average of at least four technical replicates per experimental condition, reflecting consistent trends seen across biological replicates.

### 4.4. Fast Repetition Rate (FRR) Fluorometry and Q_A_^−^ Redox Kinetics

Variable chlorophyll *a* fluorescence (F_v_) was measured using a SpectroLogix JTS-150 (Knoxville, TN, USA) spectrometer adapted for fast repetition rate fluorometry (FRR) [57,90,91]. The maximum quantum yield of PSII was calculated as F_v_/F_m_, where F_v_ = F_m_ − F_o_; F_o_ represents the initial fluorescence and F_m_ represents the maximal saturated fluorescence [57]. To probe S-state transitions within the water oxidizing complex, STFs were delivered via a 5-watt TTL controlled laser (636 nm, 24.5 µs pulse duration; RPMC Lasers, O’Fallon, MO, USA). These flashes provided sufficient energy to advance the WOC through a single transition while preventing multiple excitations within a single turnover. The resulting oscillations in F_v_/F_m_ were analyzed using a nonlinear least-squares fitting based on the VZAD algorithm, consisting of a modified Joliot–Kok model tailored for WOC kinetics [92,93]. Model accuracy was verified by the root mean square deviation (RMSD) between experimental data and the calculated fit. Reported FRR parameters were derived from the mean of 15 flash trains (50 flashes each), a measurement series representative of 10 technical and/or biological replicates. Y_3_–Y_5_ values were obtained from these FRR measurements [57].

Q_A_^−^ reoxidation kinetics were measured using a pump-probe flash protocol on the same fluorometer. Fluorescence emission from reaction center chlorophyll *a* (P680) within PSII increases when forward electron transfer from the semi-stable acceptor, Q_A_, to the secondary mobile acceptor, Q_B_, is delayed [3,4,5]. Simultaneously, the rate at which P680^+^ is reduced depends on the functional stability of the donor side, specifically the water oxidizing complex [11,40,44,66]. Following a 120 s dark adaptation, cells were subjected to a saturating pump flash, followed by subsequent probe flashes at varying intervals to monitor the fluorescence decay from F_m_ to F_o_. The resulting decay curves were fitted to a biphasic exponential model to calculate Q_A_^−^ reoxidation rates [94]. Values reported are the average of at least 4 independent biological replicates.

### 4.5. Absorbance Redox Kinetic Measurements

The redox dynamics of the cytochrome *b_6_f* complex and PSI were monitored using the SpectroLogix JTS-150 spectrometer [95,96]. Photo-stimulation was provided by actinic light at 630 nm. To quantify the oxidation states of the heme centers and copper binding sites, differential absorbance measurements were recorded at 546 nm (cytochrome b), 554 nm (cytochrome f), and 574 nm (plastocyanin). A multiple bandpass filter (Schott BG-39; Edmund Optics, Barrington, NJ, USA) was employed to facilitate concurrent data collection across all wavelengths. PSI redox kinetics were assessed by monitoring the photo-oxidation of the P700 reaction center. Measurements were performed using a specialized P700 filter set, tracking the absorbance at 810 nm (the primary P700 ground state band) alongside scattering controls at 705 nm and 740 nm [97,98]. All absorbance kinetics for both cytochrome *b_6_f* and P700 represent the average of at least 4 biological replicates.

### 4.6. Low-Temperature (77 K) Fluorescence Spectroscopy

The efficiency of light harvesting and excitonic energy distribution was measured via steady-state fluorescence emission at 77 K [99]. Measurements were performed using a JASCO FP-8300 fluorometer (Tokyo, Japan) equipped with a liquid nitrogen closed sample assembly (PMU-830). Cooling samples to 77 K is a standard technique to freeze biochemical transients and prevent protein reorganization, allowing for the clear spectral resolution of Photosystem I (PSI) and Photosystem II (PSII) emission bands [86,100]. Samples were excited at pigment-specific wavelengths: chlorophyll was excited at 435 nm using a scan speed of 200 nm/min, while phycobilins were excited at 561 nm with a scan speed of 500 nm/min. Emission spectra were recorded between 580 nm and 750 nm. To facilitate comparison across treatments, all spectra were normalized to the PSI fluorescence maximum (approximately 730 nm). This method allowed for the assessment of antenna connectivity and photosystem stoichiometry, as well as partitioning of energy delivery within PSII between F685 and F695 [101,102]. According to prior studies, the F695 signal (occurring between 694–697 nm) represents excitons that have become trapped on a specific red-absorbing chlorophyll in CP47, and this emission typically originates from centers that are unable to advance those excitons to P680 [86]. The experiment was performed with five biological replicates, each containing three technical replicates. The data represent the consistent spectral trends observed across all replicates.

## 5. Conclusions

The findings of this study demonstrate the relationship between chloride and bicarbonate in maintaining the functional integrity of Photosystem II. While these cofactors are often discussed as separate components, these results from *L. maxima* demonstrate that their roles in proton-coupled electron transfer and regulation of excitation energy are linked. This interaction is not limited to long-range feedback from the acceptor side, but rather the data indicate that bicarbonate participation is also localized to the donor side despite this depletion method being better known as an acceptor-side treatment. In bromide-substituted cells, bicarbonate depletion results in a suppression of S-state transitions. The failure of these centers to fully recover following bicarbonate repletion, demonstrated by the permanent loss in Y_3_–Y_5_ amplitude and steady-state oxygen yields, reveals that chloride is required to maintain the structural integrity of the water oxidizing complex during stress. These findings are consistent with the model in which bicarbonate functions as a proton acceptor in coordination with the chloride-dependent proton egress pathway. Under this interpretation, chloride provides the structural gating necessary to maintain the proton channels, while bicarbonate facilitates the chemical buffering required for efficient S-state transitions. The system maintains energetic uncoupling until the core is stable; in the bromide-substituted environment, reintroduction of bicarbonate is followed by phycobilisome recoupling while the WOC remains kinetically impaired. This leads to the accumulation of excitation energy at the CP47 trap, indicating that the reaction center has become an energetic trap. Ultimately, these results show that chloride and bicarbonate function in a coordinated manner to maintain electron transport and prevent photoinhibition. This study highlights the specific inorganic cofactor interactions required to maintain photosynthetic efficiency and prevent damage.

## Figures and Tables

**Table 1:** Rate of oxygen evolution, activity loss, and total recovery across halide and bicarbonate treatments. Measurements were obtained via Clark-type electrodes to determine the rates of net oxygen production (evolution) and dark respiration (consumption). Values are presented for chloride and bromide cells across control, bicarbonate-depleted and bicarbonate-repleted conditions. Data represent the average of at least four biological replicates, with rates normalized to chlorophyll *a* concentration.

Condition	Chloride (µmol O_2_ mg Chl *a*^−1^ h^−1^)	Bromide (µmol O_2_ mg Chl *a*^−1^ h^−1^)
Control	113.3 ± 4.99	106.3 ± 6.28
Depleted (no HCO_3_^−^)	46.21 ± 14.89	30.44 ± 3.36
Repleted	118.1 ± 14.95	87.86 ± 6.25
Activity Loss	59.20 ± 13.43%	71.35 ± 5.15%
Total Recovery	104.3 ± 13.98%	82.68 ± 7.64%

**Table 2:** Inefficiency and S-state populations derived from the VZAD model across halide and bicarbonate treatments. Fits were obtained from flash-induced fluorescence oscillations for chloride and bromide cells in control, bicarbonate-depleted and-repleted conditions. Data include inefficiency parameters and the distribution of S-states.

Inefficiency Parameters and S-State Populations					
Parameters	Cl^−^	Cl^−^, No HCO_3_^−^	Cl^−^, Repletion	Br^−^	Br^−^, Repletion
α	0.125	0.109	0.205	0.179	0.194
β	0.047	0.096	0.000	0.000	0.000
δ	0.005	0.000	0.025	0.003	0.071
S_0_	0.284	0.145	0.000	0.073	0.000
S_1_	0.386	0.524	0.419	0.427	0.459
S_2_	0.330	0.331	0.581	0.500	0.541
S_3_	0.000	0.000	0.000	0.000	0.000
RMSE	2.71%	6.10%	6.64%	3.03%	14.5%

**Table 3:** Kinetic parameters of Q_A_^−^ reoxidation derived from biphasic exponential decay fits. Values shown represent chloride and bromide cultures across control, depleted, and repleted states. y_0_ represents the offset (fraction of centers not active); A_1_ and t_1_ represent the amplitude and time constant for electron transfer from Q_A_^−^ to Q_B_, respectively; A_2_ and t_2_ represent the equivalent parameters for transfer from Q_A_^−^ to Q_B_^−^.

Q_A_^−^ Reoxidation Parameters						
Parameters	Chloride	Bromide	Chloride Depleted 5-Point	Bromide Depleted 5-Point	Chloride Repletion	Bromide Repletion
y_0_	0.561 ± 0.005	0.565 ± 0.031	0.767 ± 0.016	0.716 ± 0.010	0.596 ± 0.005	0.543 ± 0.018
A_1_	0.158 ± 0.010	0.220 ± 0.024	0.211 ± 0.016	0.297 ± 0.014	0.220 ± 0.016	0.286 ± 0.017
t_1_, µs	158 ± 14.3	103 ± 23.5	72.6.0 ± 17.9	52.90 ± 7.22	171 ± 16.3	153 ± 14.8
A_2_	0.234 ± 0.008	0.207 ± 0.023	-	-	0.157 ± 0.015	0.149 ± 0.013
t_2_, µs	1420 ± 130	1850 ± 810	-	-	1160 ± 190	1970 ± 740

**Table 4:** The 77 K emission peak ratios (F685:F695 and PSII:PSI) derived from chlorophyll excitation at 435 nm. Values are shown for *Limnospira maxima* across native control, bicarbonate-depleted, and bicarbonate-repleted conditions in both chloride and bromide-substituted cells. Data represent the representative dataset from at least six independent biological replicates (*n* = 6), each measured in technical triplicates.

*L. maxima* Chlorophyll Area Ratios		
Condition	F685:F695	PSII:PSI
Cl^−^	0.235 ± 0.023	0.270 ± 0.007
Cl^−^, no HCO_3_^−^	0.381 ± 0.040	0.193 ± 0.008
Cl-, repleted	0.447 ± 0.033	0.209 ± 0.006
Br^−^	0.356 ± 0.017	0.311 ± 0.006
Br^−^, no HCO_3_^−^	0.437 ± 0.027	0.249 ± 0.006
Br^−^, repleted	0.308 ± 0.015	0.299 ± 0.005

**Table 5:** The 77 K fluorescence emission peak ratios (CPC:APC, F685:F695, PSII:PSI and PB:PS) derived from phycobilin excitation at 561 nm. Values shown across native control, bicarbonate-depleted, and bicarbonate-repleted conditions in both chloride and bromide-substituted *L. maxima* cells. Data shown represent the average of six biological replicates (*n* = 6), each measured in technical triplicate.

*L. maxima* Phycobilin Area Ratios				
	CPC:APC	F685:F695	PSII:PSI	PB:PS
Cl^−^	1.31 ± 0.06	0.140 ± 0.033	0.928 ± 0.046	0.572 ± 0.019
Cl^−^, no HCO_3_^−^	1.14 ± 0.07	0.121 ± 0.071	1.00 ± 0.11	0.738 ± 0.046
Cl^−^, repleted	1.07 ± 0.07	0.104 ± 0.036	1.02 ± 0.07	0.607 ± 0.029
Br^−^	1.10 ± 0.07	0.089 ± 0.04	0.955 ± 0.057	0.720 ± 0.031
Br^−^, no HCO_3_^−^	1.14 ± 0.09	0.179 ± 0.127	1.01 ± 0.17	0.809 ± 0.074
Br^−^, repleted	1.06 ± 0.11	0.084 ± 0.034	1.06 ± 0.07	0.643 ± 0.040

## Data Availability

The original contributions presented in this study are included in the article/Supplementary Material. Further inquiries can be directed to the corresponding author.

## Supplementary Materials

The following supporting information can be downloaded at: https://www.mdpi.com/article/10.3390/plants15101490/s1, Figure S1: Individual biphasic exponential decay fit curves of Q_A_^−^ reoxidation across halide, bicarbonate-depleted and-repleted treatments; Figure S2: Gaussian deconvolution of 77 K chlorophyll fluorescence emission spectra for *L. maxima* (native chloride); Table S1: Fluorescence parameters derived from Gaussian deconvolution of 77 K spectra for native *L. maxima*; Figure S3: Gaussian deconvolution of 77 K chlorophyll fluorescence emission spectra for *L. maxima* (native chloride, bicarbonate-depleted); Table S2: Fluorescence parameters derived from Gaussian deconvolution of 77 K spectra for native bicarbonate-depleted *L. maxima*; Figure S4: Gaussian deconvolution of 77 K chlorophyll fluorescence emission spectra for *L. maxima* (native chloride, bicarbonate-repleted); Table S3: Fluorescence parameters derived from Gaussian deconvolution of 77 K spectra for native bicarbonate-repleted *L. maxima*; Figure S5: Gaussian deconvolution of 77 K chlorophyll fluorescence emission spectra for *L. maxima* (bromide); Table S4: Fluorescence parameters derived from Gaussian deconvolution of 77 K spectra for bromide *L. maxima*; Figure S6: Gaussian deconvolution of 77 K chlorophyll fluorescence emission spectra for *L. maxima* (bromide, bicarbonate-depleted); Table S5: Fluorescence parameters derived from Gaussian deconvolution of 77 K spectra for bromide bicarbonate-depleted *L. maxima*; Figure S7: Gaussian deconvolution of 77 K chlorophyll fluorescence emission spectra for *L. maxima* (bromide, bicarbonate-repleted); Table S6: Fluorescence parameters derived from Gaussian deconvolution of 77 K spectra for bromide bicarbonate-repleted *L. maxima*; Figure S8: Gaussian deconvolution of 77 K phycobilin fluorescence emission spectra for *L. maxima* (native chloride); Table S7: Fluorescence parameters derived from Gaussian deconvolution of 77 K spectra for native *L. maxima*; Figure S9: Gaussian deconvolution of 77 K phycobilin fluorescence emission spectra for *L. maxima* (native chloride, bicarbonate-depleted); Table S8: Fluorescence parameters derived from Gaussian deconvolution of 77 K spectra for native bicarbonate-depleted *L. maxima*; Figure S10: Gaussian deconvolution of 77 K phycobilin fluorescence emission spectra for *L. maxima* (native chloride, bicarbonate-repleted); Table S9: Fluorescence parameters derived from Gaussian deconvolution of 77 K spectra for native bicarbonate-repleted *L. maxima*; Figure S11: Gaussian deconvolution of 77 K phycobilin fluorescence emission spectra for *L. maxima* (bromide); Table S10: Fluorescence parameters derived from Gaussian deconvolution of 77 K spectra for bromide *L. maxima*; Figure S12: Gaussian deconvolution of 77 K phycobilin fluorescence emission spectra for *L. maxima* (bromide, bicarbonate-depleted); Table S11: Fluorescence parameters derived from Gaussian deconvolution of 77 K spectra for bromide bicarbonate-depleted *L. maxima*; Figure S13: Gaussian deconvolution of 77 K phycobilin fluorescence emission spectra for *L. maxima* (bromide, bicarbonate-repleted); Table S12: Fluorescence parameters derived from Gaussian deconvolution of 77 K spectra for bromide bicarbonate-repleted *L. maxima*.

## Abbreviations

The following abbreviations are used in this manuscript:

PSII	Photosystem II
WOC	Water-oxidizing complex
PCET	Proton-coupled electron transfer
Cryo-EM	Cryo-electron microscopy
D1, D2, CP43, and CP47	Subunits of Photosystem II
NHI	Non-heme iron
Q_A_ and Q_B_	The primary and secondary plastoquinone electron acceptors in Photosystem II
PQ	Plastoquinone
Chl	Chlorophyll
FRR	Fast Repetition Rate (fluorometry)
VZAD	Vinyard, Zachary, Ananyev, and Dismukes matrix model of WOC cycling
STF	Single turnover flash, enough light to produce one charge separation in all PSII in a sample
RMSE	Root mean square error
Y_3_ and Y_5_	Yield on flashes 3 and 5 of a series of STFs
PSI	Photosystem I
PC	Plastocyanin
cyt	Cytochrome
77 K	77 degrees Kelvin, i.e., liquid nitrogen temperature for spectrofluorometry
F685 and F695	Fluorescence emission at 685 and 695 nm
CPC	c-phycocyanin
APC	Allophycocyanin
PB	Phycobilin
PS	Photosystem
P680	Reaction center of Photosystem II
P700	Reaction center of Photosystem I
UTEX	University of Texas at Austin Culture Collection
LED	Light-emitting diode
TTL	Transistor-transistor logic
Fv	Chlorophyll variable fluorescence
Fm	Chlorophyll maximal fluorescence
Fo	Chlorophyll initial fluorescence
RMSD	Root mean square deviation
