# Supplementary material for: Interrelated Roles of Chloride and Bicarbonate in Regulating Electron Transport Across Photosystem II in Limnospira maxima
Source: Plants (Basel). 2026 May 13;15(10):1490. doi: 10.3390/plants15101490 (PMC13211245; doi:10.3390/plants15101490)
Supplement: Supplementary file 1 [file plants-15-01490-s001.zip › plants-4287920-supplementary.pdf]

Supporting Information for: Interrelated Roles of Chloride and Bicarbonate in Regulating Electron Transport Across Photosystem II in *Limnospira maxima*

Authors: Leslie Castillo[1], Nicole Seliga[1], Nidhi Patel[1], Grant Steiner[1], Gustavo Chavez[1], Alexis Diaz[1] and Colin Gates[1,2]

Affiliations: [1]Department of Chemistry and Biochemistry, [2]Department of Biology, and [3]Department of Bioinformatics, Loyola University Chicago, 1068 W. Sheridan Rd. 60660

Corresponding Author: Colin Gates, cgates4@luc.edu

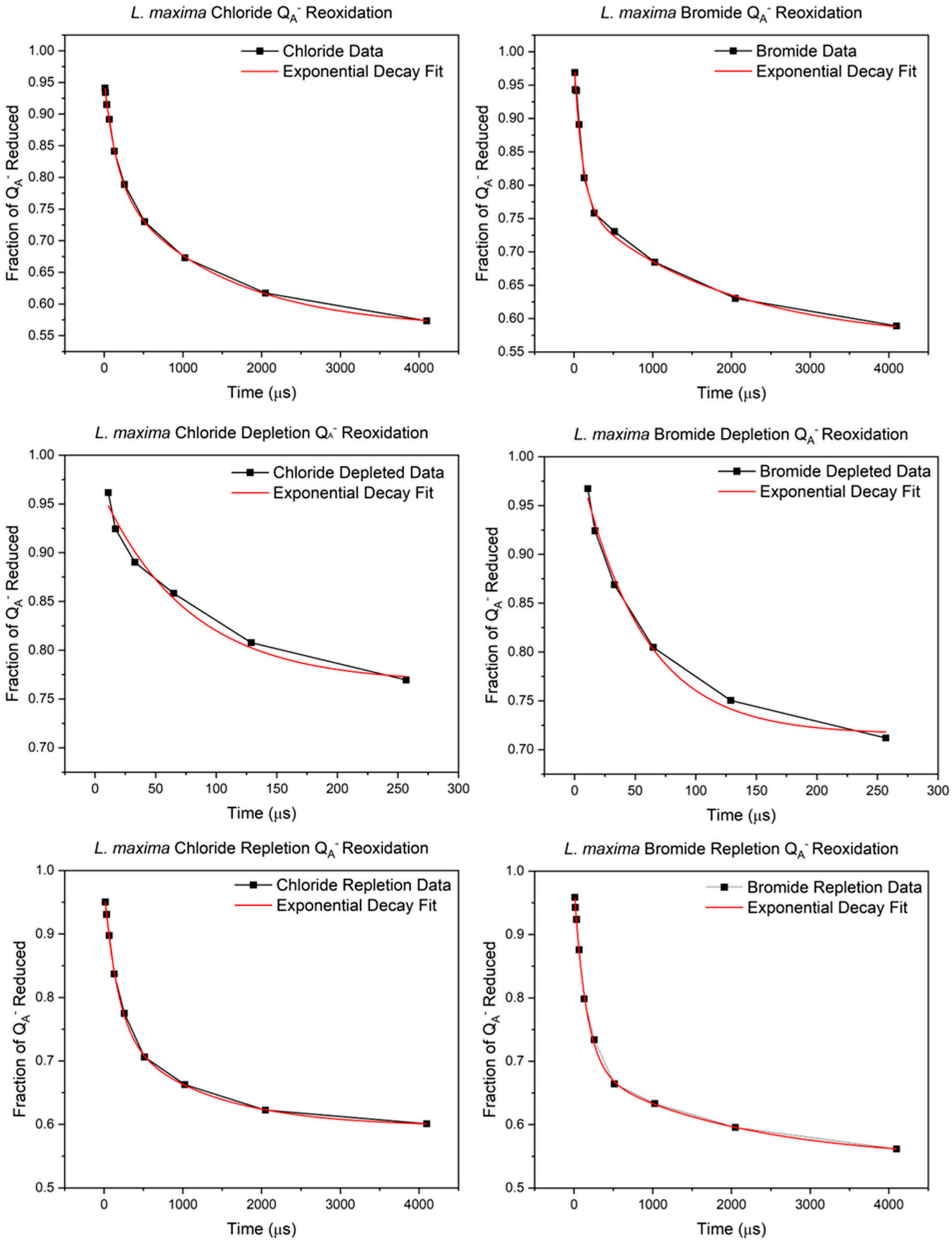
L. maxima Chloride Q$_A^-$ Reoxidation
Chloride Data
Exponential Decay Fit
Fraction of Q$_A^-$ Reduced
Time (μs)
L. maxima Bromide Q$_A^-$ Reoxidation
Bromide Data
Exponential Decay Fit
Fraction of Q$_A^-$ Reduced
Time (μs)
L. maxima Chloride Depletion Q$_A^-$ Reoxidation
Chloride Depleted Data
Exponential Decay Fit
Fraction of Q$_A^-$ Reduced
Time (μs)
L. maxima Bromide Depletion Q$_A^-$ Reoxidation
Bromide Depleted Data
Exponential Decay Fit
Fraction of Q$_A^-$ Reduced
Time (μs)
L. maxima Chloride Repletion Q$_A^-$ Reoxidation
Chloride Repletion Data
Exponential Decay Fit
Fraction of Q$_A^-$ Reduced
Time (μs)
L. maxima Bromide Repletion Q$_A^-$ Reoxidation
Bromide Repletion Data
Exponential Decay Fit
Fraction of Q$_A^-$ Reduced
Time (μs)

Figure S1. Individual biphasic exponential decay fit curves of Q_A^- reoxidation across halide, bicarbonate depleted and repleted treatments.

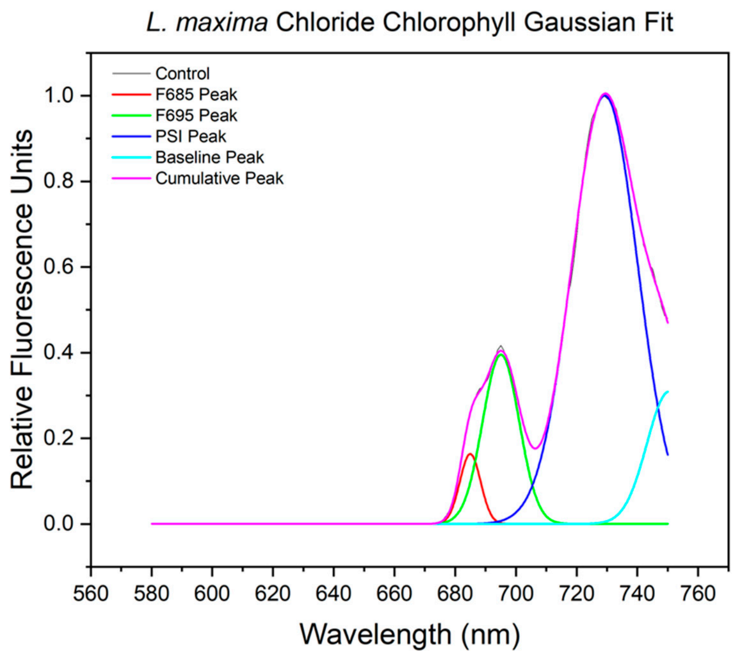

Figure S2. Gaussian deconvolution of 77K chlorophyll fluorescence emission spectra for *L. maxima* (native chloride). To calculate emission ratios, the relative areas of peaks corresponding to F685, F695, and PSI were determined through gaussian fitting. The total PSII emission area was defined as the sum of the F685 and F695 components.

Gaussian Fit of Control Chlorophyll Emission at 77K of *L. maxima*				
Plot	F685 Peak	F695 Peak	PSI Peak	Background
Center (nm)	684.95 ± 0.16	695.06 ± 0.15	729.25 ± 0.14	750.47± 0.44
Area	1.40 ± 0.13	5.9594 ± 0.15	27.22 ± 0.33	5.68 ± 0.66
Adj. R-Square	0.9997			

Table S1. Fluorescence parameters derived from Gaussian deconvolution of 77K spectra for native *L. maxima*. PSII:PSI and F685:F695 ratios were calculated based on the fitted peak areas of individual chlorophyll emission components.

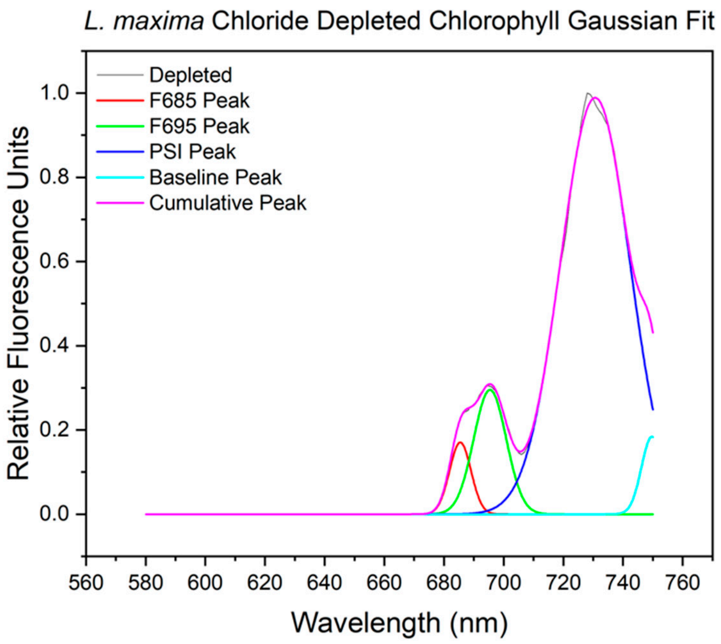

Figure S3. Gaussian deconvolution of 77K chlorophyll fluorescence emission spectra for chloride-grown bicarbonate depleted *L. maxima*. To calculate emission ratios, the relative areas of peaks corresponding to F685, F695, and PSI were determined through gaussian fitting. The total PSII emission area was defined as the sum of the F685 and F695 components.

**Gaussian Fit of Chloride Depleted Chlorophyll Emission at 77K of *L. maxima***				
Plot	F685 Peak	F695 Peak	PSI Peak	Baseline Peak
Center (nm)	685.45 ± 0.23	695.41 ± 0.23	730.66 ± 0.04	749.68 ± 0.34
Area	1.54 ± 0.15	4.04 ± 0.16	28.84 ± 0.12	1.61 ± 0.18
Adj. R-Square	0.9995			

Table S2. Fluorescence parameters derived from Gaussian deconvolution of 77K spectra for chloride-grown bicarbonate depleted *L. maxima.* PSII:PSI and F685:F695 ratios were calculated based on the fitted peak areas of individual chlorophyll emission components.

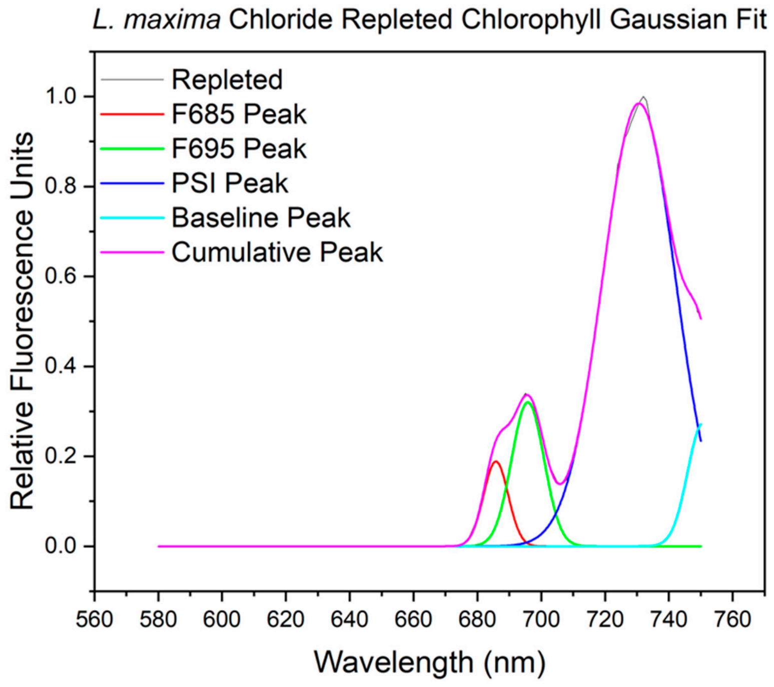

Figure S4. Gaussian deconvolution of 77K chlorophyll fluorescence emission spectra for chloride-grown bicarbonate repleted *L. maxima*. To calculate emission ratios, the relative areas of peaks corresponding to F685, F695, and PSI were determined through gaussian fitting. The total PSII emission area was defined as the sum of the F685 and F695 components.

**Gaussian Fit of Chloride Repleted Chlorophyll Emission at 77K of *L. maxima***				
Plot	F685 Peak	F695 Peak	PSI Peak	Baseline Peak
Center (nm)	685.78 ± 0.20	695.78 ± 0.17	730.63 ± 0.05	750.73 ± 0.38
Area	1.82 ± 0.12	4.08 ± 0.13	28.20 ± 0.12	3.33 ± 0.30
Adj. R-Square	0.9997			

Table S3. Fluorescence parameters derived from Gaussian deconvolution of 77K spectra for chloride-grown bicarbonate repleted *L. maxima.* PSII:PSI and F685:F695 ratios were calculated based on the fitted peak areas of individual chlorophyll emission components.

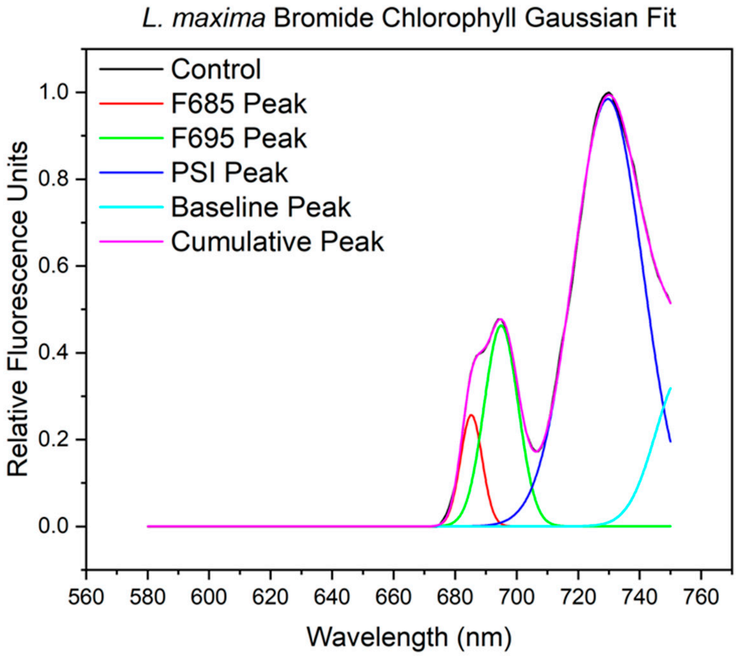

Figure S5. Gaussian deconvolution of 77K chlorophyll fluorescence emission spectra for bromide substituted *L. maxima*. To calculate emission ratios, the relative areas of peaks corresponding to F685, F695, and PSI were determined through gaussian fitting. The total PSII emission area was defined as the sum of the F685 and F695 components.

**Gaussian Fit of Bromide Chlorophyll Emission at 77K of *L. maxima***				
Plot	F685 Peak	F695 Peak	PSI Peak	Baseline Peak
Center (nm)	685.25 ± 0.09	694.96 ± 0.10	729.68 ± 0.16	754.16 ± 1.09
Area	2.28 ± 0.10	6.40 ± 0.11	27.88 ± 0.39	7.99 ± 1.40
Adj. R-Square	0.9998			

Table S4. Fluorescence parameters derived from Gaussian deconvolution of 77K spectra for bromide substituted *L. maxima.* PSII:PSI and F685:F695 ratios were calculated based on the fitted peak areas of individual chlorophyll emission components.

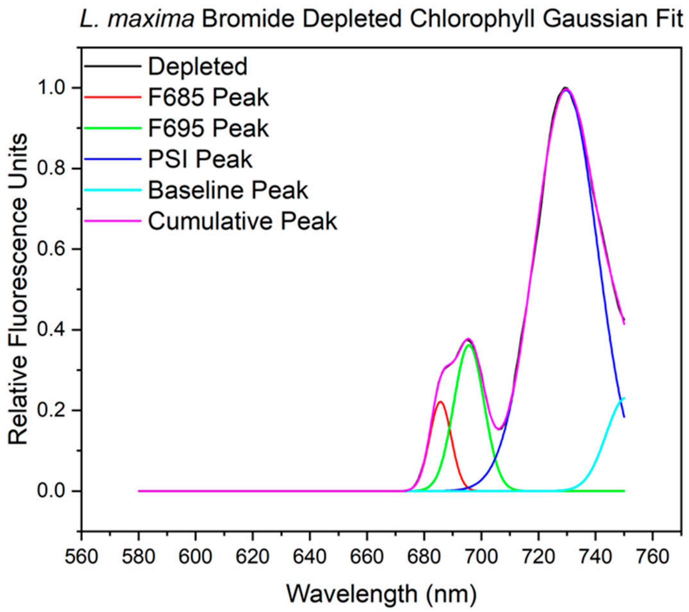

Figure S6. Gaussian deconvolution of 77K chlorophyll fluorescence emission spectra for bromide-grown bicarbonate depleted *L. maxima*. To calculate emission ratios, the relative areas of peaks corresponding to F685, F695, and PSI were determined through gaussian fitting. The total PSII emission area was defined as the sum of the F685 and F695 components.

**Gaussian Fit of Bromide Depleted Chlorophyll Emission at 77K of *L. maxima***				
Plot	F685 Peak	F695 Peak	PSI Peak	Baseline Peak
Center (nm)	685.69 ± 0.15	695.67 ± 0.14	729.66 ± 0.10	750.69 ± 0.51
Area	2.09 ± 0.12	4.79 ± 0.12	27.58 ± 0.24	4.08 ± 0.50
Adj. R-Square	0.9998			

Table S5. Fluorescence parameters derived from Gaussian deconvolution of 77K spectra for bromide-grown bicarbonate depleted *L. maxima.* PSII:PSI and F685:F695 ratios were calculated based on the fitted peak areas of individual chlorophyll emission components.

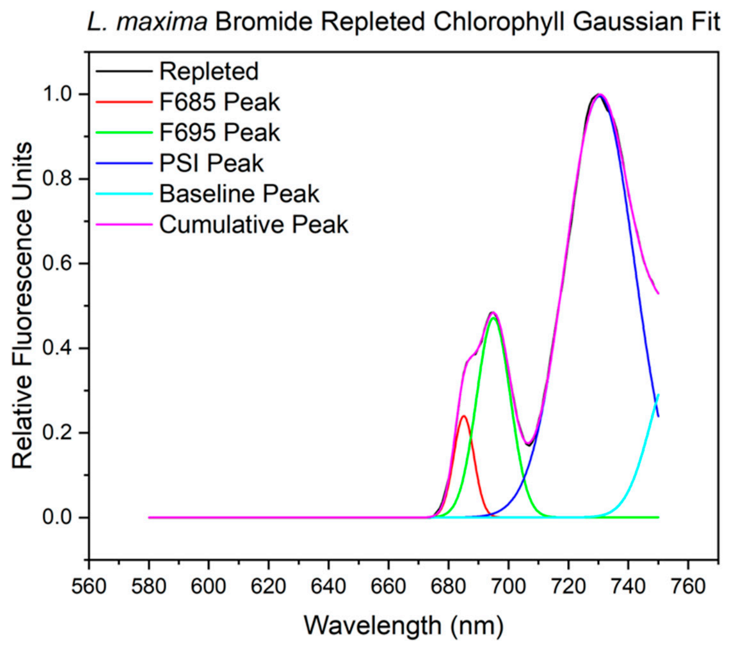

Figure S7. Gaussian deconvolution of 77K chlorophyll fluorescence emission spectra for bromide-grown bicarbonate repleted *L. maxima*. To calculate emission ratios, the relative areas of peaks corresponding to F685, F695, and PSI were determined through gaussian fitting. The total PSII emission area was defined as the sum of the F685 and F695 components.

**Gaussian Fit of Bromide Repleted Chlorophyll Emission at 77K of *L. maxima***				
Plot	F685 Peak	F695 Peak	PSI Peak	Baseline Peak
Center (nm)	685.09 ± 0.09	695.02 ± 0.09	730.43 ± 0.12	755.84 ± 1.83
Area	2.04 ± 0.09	6.62 ± 0.10	28.90 ± 0.29	7.78 ± 1.96
Adj. R-Square	0.9998			

Table S6. Fluorescence parameters derived from Gaussian deconvolution of 77K spectra for bromide-bicarbonate repleted *L. maxima.* PSII:PSI and F685:F695 ratios were calculated based on the fitted peak areas of individual chlorophyll emission components.

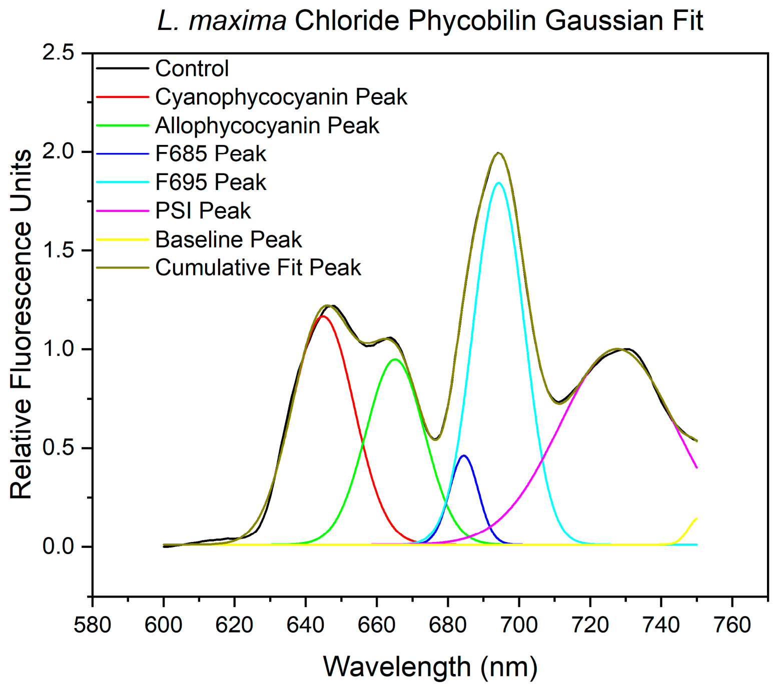

Figure S8. Gaussian deconvolution of 77K phycobilin fluorescence emission spectra for native *L. maxima* (native chloride). Spectra were resolved into individual peaks for CPC, APC, F685, F695, and PSI. The resulting integrated peak areas were used to calculate emission ratios (CPC:APC, F685:F695, PSII:PSI, and PB:PS). For these calculations total PSII emission was defined as the sum of the F685 and F695. The phycobilisome (PB) area was calculated as the sum of CPC and APC components, while the total photosystems (PS) area was defined as the combined PSII and PSI.

**Gaussian Fit of Chloride Phycobilin Emission at 77K of *L. maxima***						
Plot	CPC Peak	APC Peak	F685 Peak	F695 Peak	PSI Peak	Baseline Peak
Center (nm)	644.88 ± 0.24	665.16 ± 0.26	684.48 ± 0.25	694.29 ± 0.25	727.66 ± 0.15	750.67 ± 2.16
Area	25.37 ± 0.74	19.43 ± 0.76	4.61 ± 1.09	33.06 ± 1.36	40.61 ± 0.73	1.03 ± 0.73
Adj. R-Square	0.9987					

Table S7. Emission peak parameters for native *L. maxima* derived from Gaussian fitting of 77 K phycobilin fluorescence spectra. Integrated areas for c-phycocyanin (CPC), allophycocyanin (APC), F685, F695, and PSI were used to determine relative emission ratios. Total phycobilisome (PB) emission was calculated as the sum of CPC and APC areas, while total photosystem (PS) emission was defined as the combined area of the PSII (F685 and F695) and PSI components.

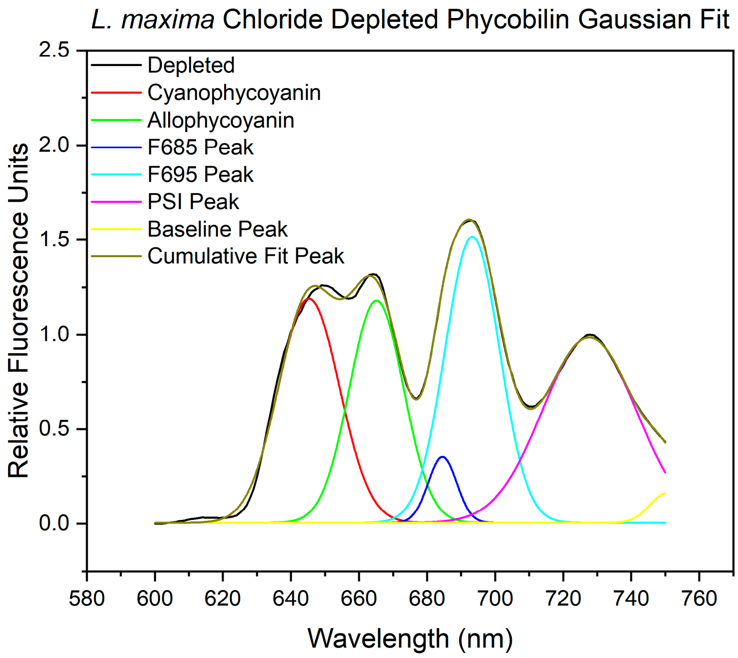

Figure S9. Gaussian deconvolution of 77K phycobilin fluorescence emission spectra for chloride-grown bicarbonate depleted *L. maxima*. Spectra were resolved into individual peaks for CPC, APC, F685, F695, and PSI. The resulting integrated peak areas were used to calculate emission ratios (CPC:APC, F685:F695, PSII:PSI, and PB:PS). For these calculations total PSII emission was defined as the sum of the F685 and F695. The phycobilisome (PB) area was calculated as the sum of CPC and APC components, while the total photosystems (PS) area was defined as the combined PSII and PSI.

**Gaussian Fit of Chloride Depleted Phycobilin Emission at 77K of *L. maxima***						
Plot	CPC Peak	APC Peak	F685 Peak	F695 Peak	PSI Peak	Baseline Peak
Center (nm)	645.28 ± 0.32	665.13 ± 0.24	684.52 ± 0.30	693.29 ± 0.67	727.67 ± 0.21	750.60 ± 2.72
Area	26.71 ± 0.97	23.49 ± 1.14	3.68 ± 2.13	30.35 ± 2.83	33.95 ± 1.03	2.00 ± 1.36
Adj. R-Square	0.9981					

Table S8. Emission peak parameters for chloride-grown bicarbonate depleted *L. maxima* derived from Gaussian fitting of 77 K phycobilin fluorescence spectra. Integrated areas for c-phycocyanin (CPC), allophycocyanin (APC), F685, F695, and PSI were used to determine relative emission

ratios. Total phycobilisome (PB) emission was calculated as the sum of CPC and APC areas, while total photosystem (PS) emission was defined as the combined area of the PSII (F685 and F695) and PSI components.

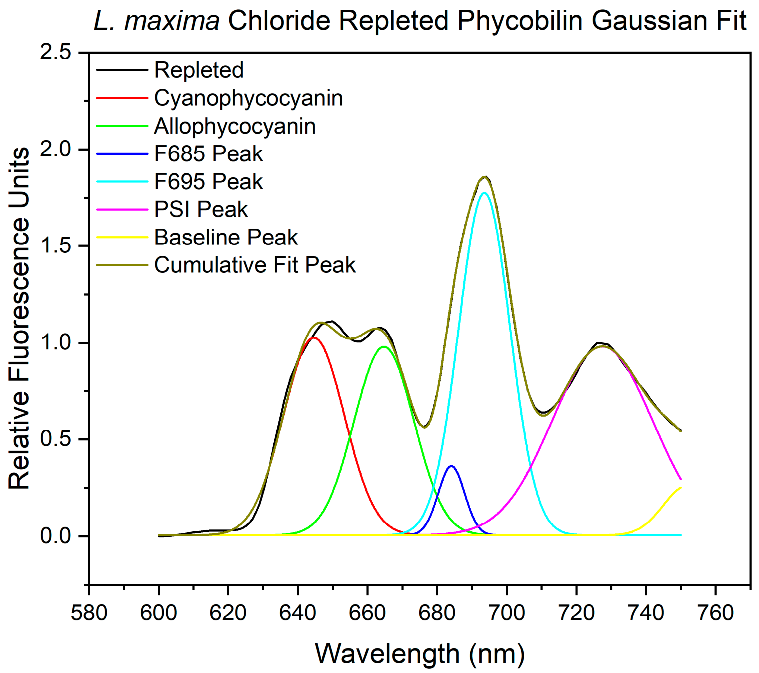

Figure S10. Gaussian deconvolution of 77K phycobilin fluorescence emission spectra for chloride-grown bicarbonate repleted *L. maxima*. Spectra were resolved into individual peaks for CPC, APC, F685, F695, and PSI. The resulting integrated peak areas were used to calculate emission ratios (CPC:APC, F685:F695, PSII:PSI, and PB:PS). For these calculations total PSII emission was defined as the sum of the F685 and F695. The phycobilisome (PB) area was calculated as the sum of CPC and APC components, while the total photosystems (PS) area was defined as the combined PSII and PSI.

**Gaussian Fit of Chloride Repleted Phycobilin Emission at 77K of *L. maxima***						
Plot	CPC Peak	APC Peak	F685 Peak	F695 Peak	PSI Peak	Baseline Peak
Center (nm)	644.58 ± 0.35	664.74 ± 0.33	684.09 ± 0.27	693.57 ± 0.29	727.45 ± 0.44	751.86 ± 3.02
Area	22.40 ± 0.96	20.99 ± 1.02	3.41 ± 1.15	32.76 ± 1.64	35.31 ± 1.65	4.41 ± 2.84
Adj. R-Square	0.9982					

Table S9. Emission peak parameters for chloride-grown bicarbonate repleted *L. maxima* derived from Gaussian fitting of 77 K phycobilin fluorescence spectra. Integrated areas for c-phycocyanin (CPC), allophycocyanin (APC), F685, F695, and PSI were used to determine relative emission ratios. Total phycobilisome (PB) emission was calculated as the sum of CPC and APC areas, while total photosystem (PS) emission was defined as the combined area of the PSII (F685 and F695) and PSI components.

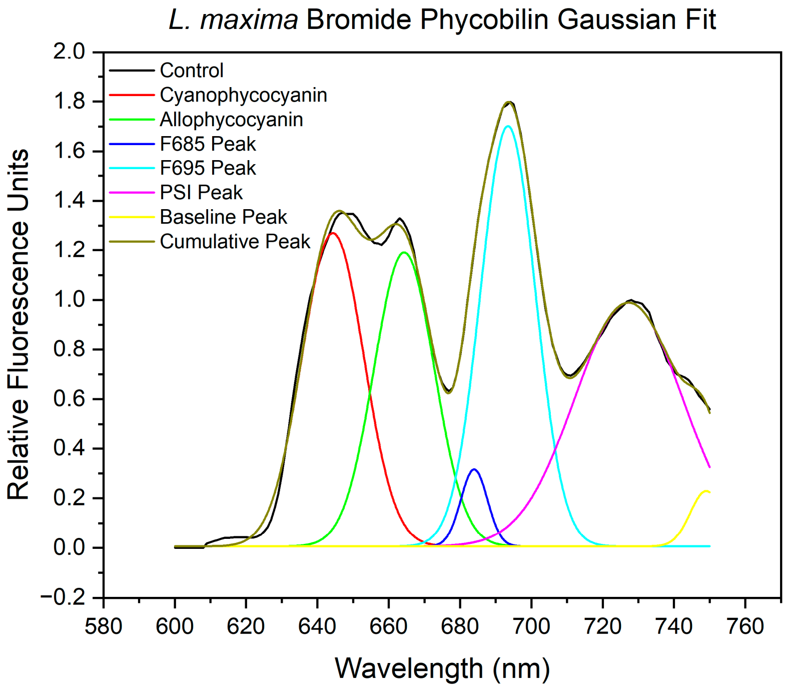

Figure S11. Gaussian deconvolution of 77K phycobilin fluorescence emission spectra for bromide substituted *L. maxima*. Spectra were resolved into individual peaks for CPC, APC, F685, F695, and PSI. The resulting integrated peak areas were used to calculate emission ratios (CPC:APC, F685:F695, PSII:PSI, and PB:PS). For these calculations total PSII emission was defined as the sum of the F685 and F695. The phycobilisome (PB) area was calculated as the sum of CPC and APC components, while the total photosystems (PS) area was defined as the combined PSII and PSI.

**Gaussian Fit of Bromide Phycobilin Emission at 77K of *L. maxima***						
Plot	CPC Peak	APC Peak	F685 Peak	F695 Peak	PSI Peak	Baseline Peak
Center (nm)	644.33 ± 0.33	664.32 ± 0.31	683.94 ± 0.31	693.43 ± 0.35	727.27 ± 0.24	749.04 ± 0.97

Area	27.49 ± 1.09	25.07 ± 1.17	2.91 ± 1.24	32.75 ± 1.87	37.36 ± 1.28	2.51 ± 0.87
Adj. R-Square	0.9976					

Table S10. Emission peak parameters for bromide substituted *L. maxima* derived from Gaussian fitting of 77 K phycobilin fluorescence spectra. Integrated areas for c-phycocyanin (CPC), allophycocyanin (APC), F685, F695, and PSI were used to determine relative emission ratios. Total phycobilisome (PB) emission was calculated as the sum of CPC and APC areas, while total photosystem (PS) emission was defined as the combined area of the PSII (F685 and F695) and PSI components.

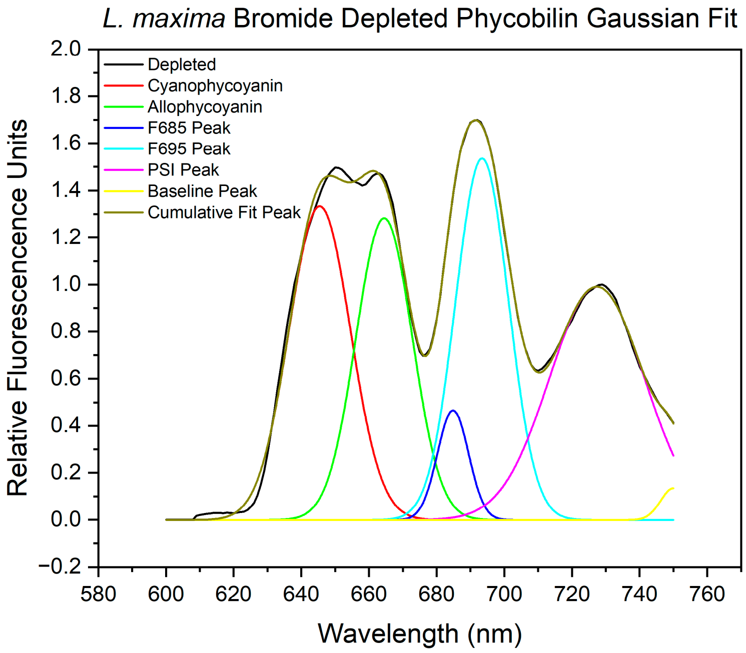

Figure S12. Gaussian deconvolution of 77K phycobilin fluorescence emission spectra for bromide-grown bicarbonate depleted *L. maxima*. Spectra were resolved into individual peaks for CPC, APC, F685, F695, and PSI. The resulting integrated peak areas were used to calculate emission ratios (CPC:APC, F685:F695, PSII:PSI, and PB:PS). For these calculations total PSII emission was defined as the sum of the F685 and F695. The phycobilisome (PB) area was calculated as the sum of CPC and APC components, while the total photosystems (PS) area was defined as the combined PSII and PSI.

Gaussian Fit of Bromide Phycobilin Emission at 77K of *L. maxima*						
Plot	CPC Peak	APC Peak	F685 Peak	F695 Peak	PSI Peak	Baseline Peak
Center (nm)	645.44 ± 0.44	664.48 ± 0.36	684.84 ± 0.40	693.43 ± 1.08	727.38 ± 0.19	750.07 ± 2.25
Area	30.36 ± 1.53	26.75 ± 1.69	5.40 ± 3.75	30.16 ± 4.46	35.04 ± 0.92	1.31 ± 0.87
Adj. R-Square	0.9978					

Figure S11. Emission peak parameters for bromide-grown bicarbonate repleted *L. maxima* derived from Gaussian fitting of 77 K phycobilin fluorescence spectra. Integrated areas for c-phycocyanin (CPC), allophycocyanin (APC), F685, F695, and PSI were used to determine relative emission ratios. Total phycobilisome (PB) emission was calculated as the sum of CPC and APC areas, while total photosystem (PS) emission was defined as the combined area of the PSII (F685 and F695) and PSI components.

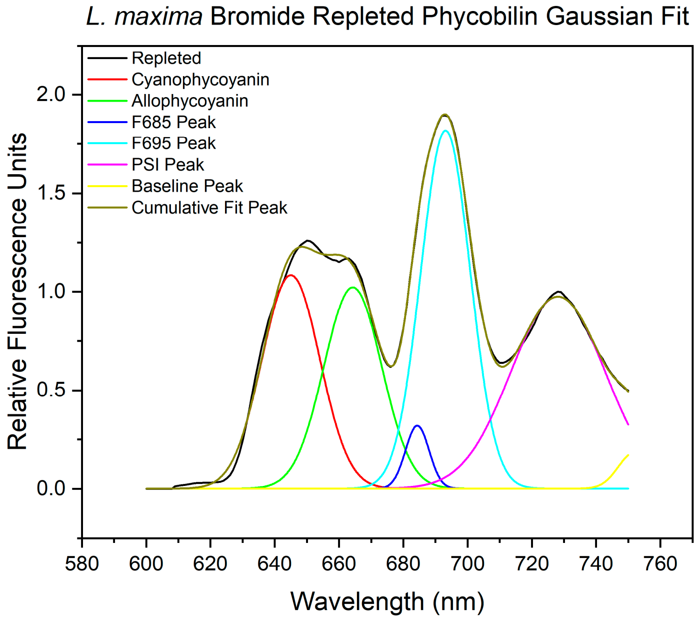

Figure S13. Gaussian deconvolution of 77K phycobilin fluorescence emission spectra for bromide-grown bicarbonate repleted *L. maxima*. Spectra were resolved into individual peaks for CPC, APC, F685, F695, and PSI. The resulting integrated peak areas were used to calculate emission ratios (CPC:APC, F685:F695, PSII:PSI, and PB:PS). For these calculations total PSII emission was defined as the sum of the F685 and F695. The phycobilisome (PB) area was

calculated as the sum of CPC and APC components, while the total photosystems (PS) area was defined as the combined PSII and PSI.

Gaussian Fit of Bromide Phycobilin Emission at 77K of *L. maxima*						
Plot	CPC Peak	APC Peak	F685 Peak	F695 Peak	PSI Peak	Baseline Peak
Center (nm)	645.03 ± 0.56	664.28 ± 0.54	684.35± 0.25	693.14 ± 0.35	728.14 ± 0.22	751.87 ± 4.25
Area	24.49 ± 1.69	23.07 ± 1.86	2.94 ± 1.20	35.08 ± 1.80	35.95 ± 1.00	2.27 ± 2.11
Adj. R-Square	0.9981					

Table S12. Emission peak parameters for bromide-grown bicarbonate repleted *L. maxima* derived from Gaussian fitting of 77 K phycobilin fluorescence spectra. Integrated areas for c-phycocyanin (CPC), allophycocyanin (APC), F685, F695, and PSI were used to determine relative emission ratios. Total phycobilisome (PB) emission was calculated as the sum of CPC and APC areas, while total photosystem (PS) emission was defined as the combined area of the PSII (F685 and F695) and PSI components.
